# Effect of Chinese Herbal Medicine Therapy on Overall and Cancer Related Mortality in Patients With Advanced Nasopharyngeal Carcinoma in Taiwan

**DOI:** 10.3389/fphar.2020.607413

**Published:** 2021-01-29

**Authors:** Chen-Yu Wang, Tang-Chuan Wang, Wen-Miin Liang, Chien-Hui Hung, Jian-Shiun Chiou, Chao-Jung Chen, Fuu-Jen Tsai, Sheng-Teng Huang, Ta-Yuan Chang, Ting-Hsu Lin, Chiu-Chu Liao, Shao-Mei Huang, Te-Mao Li, Ying-Ju Lin

**Affiliations:** ^1^ Department of Chinese Medicine, China Medical University Hospital, Taichung, Taiwan; ^2^ School of Chinese Medicine, China Medical University, Taichung, Taiwan; ^3^ Department of Public Health, China Medical University, Taichung, Taiwan; ^4^ School of Medicine, China Medical University, Taichung, Taiwan; ^5^ Department of Otolaryngology-Head and Neck Surgery, China Medical University Hsinchu Hospital, Hsinchu, Taiwan; ^6^ Department of Health Services Administration, China Medical University, Taichung, Taiwan; ^7^ Graduate Institute of Clinical Medical Sciences, Chang-Gung University, Taoyuan, Taiwan; ^8^ Division of Infectious Diseases, Chang Gung Memorial Hospital Chiayi Branch, Chiayi, Taiwan; ^9^ Genetic Center, Proteomics Core Laboratory, Department of Medical Research, China Medical University Hospital, Taichung, Taiwan; ^10^ Graduate Institute of Integrated Medicine, China Medical University, Taichung, Taiwan; ^11^ Department of Biotechnology and Bioinformatics, Asia University, Taichung, Taiwan

**Keywords:** advanced nasopharyngeal carcinoma, overall mortality, chinese herbal medicine, association rule, network analysis

## Abstract

Nasopharyngeal carcinoma (NPC) is a head and neck cancer involving epithelial squamous-cell carcinoma of the nasopharynx that mainly occurs in individuals from East and Southeast Asia. We investigated whether Chinese herbal medicine (CHM) as a complementary therapy offers benefits to these patients. We retrospectively evaluated the Taiwan Cancer Registry (Long Form) database for patients with advanced NPC, using or not using CHM, between 2007–2013. Cox proportional-hazard model and Kaplan‒Meier survival analyses were applied for patient survival. CHM-users showed a lower overall and cancer-related mortality risk than non-users. For advanced NPC patients, the overall mortality risk was 0.799-fold for CHM-users, after controlling for age, gender, and Charlson comorbidity index (CCI) score (Cancer stages 3 + 4: adjusted hazard ratio [aHR]: 0.799, 95% confidence interval [CI]: 0.676–0.943, *p* = 0.008). CHM-users also showed a lower cancer-related mortality risk than non-users (aHR: 0.71, 95% CI: 0.53–0.96, *p* = 0.0273). Association rule analysis showed that CHM pairs were Ban-Zhi-Lian (BZL; *Scutellaria barbata D.Don*) and For single herbs, Bai-Hua-She-She-Cao (Herba Hedyotis Diffusae; Scleromitrion diffusum (Willd.) R.J.Wang (syn. Hedyotis diffusa Willd.) and Mai-Men-Dong (MMD; *Ophiopogon japonicus (Thunb.) Ker Gawl.*), and Gan-Lu-Yin (GLY) and BHSSC. Network analysis revealed that BHSSC was the core CHM, and BZL, GLY, and Xin-Yi-Qing-Fei-Tang (XYQFT) were important CHMs in cluster 1. In cluster 2, ShengDH, MMD, Xuan-Shen (XS; *Scrophularia ningpoensis Hensl.*), and Gua-Lou-Gen (GLG; *Trichosanthes kirilowii Maxim.*) were important CHMs. Thus, as a complementary therapy, CHM, and particularly the 8 CHMs identified, are important for the treatment of advanced NPC patients.

## Introduction

Nasopharyngeal carcinoma (NPC) is a head and neck cancer involving epithelial squamous-cell carcinoma of the nasopharynx (Ferlay et al., 2019), which mainly occurs in individuals from East and Southeast Asia (Cao et al., 2011; Ferlay et al., 2019). The global incidence of NPC is less than 1 per 100,000 person-years; however, in Taiwan, its incidence is 2.8–6.6 per 100,000 person-years (Hsu et al., 2011; Fan et al., 2018). Furthermore, in Taiwan, NPC is the most and second most common head and neck cancer in males and females, respectively (Huang et al., 2015). NPC treatment involves integration of radiotherapy, chemotherapy, and surgery (Perri et al., 2019). The main therapies for NPC are radiotherapy alone for early stage (T1-N0M0 stage) or combined with both chemotherapy and radiotherapy for advanced stages (T2N0‒T4N3M0) (Perri et al., 2019). With radiotherapy alone or chemotherapy in patients with early or advanced NPC, the 5-years survival rate approaches 90% (Blanchard et al., 2015). However, it may cause complications (Langendijk et al., 2008; Jensen et al., 2010), such as mucositis, dermatitis, xerostomia, dysphagia, hyposalivation, xerostomia, radiation caries, sensorineural hearing loss, radioactive osteonecrosis, triceps, temporal lobe injury, and hypothyroidism. Additionally, 8–10% of these patients develop therapeutic resistance and have recurrent disease and distant metastasis (Perri et al., 2011).

There is a need for alternative therapies that can be used in combination with conventional therapies (Salehi et al., 2019a; Salehi et al., 2019b). Chinese herbal medicine (CHM) is cost-effective and has relatively few side effects over long-term usage, and patients with cancer may choose CHM as their integrative, alternative, and complementary therapy to reduce complications from conventional therapies and to improve the overall survival rate in Taiwan (Ye et al., 2015; Hung et al., 2017; Kuo et al., 2018; Li et al., 2018). CHM shows anti-cancer activity via multiple specific targets, synergistic interactions with chemotherapy drugs, and minimal, acceptable side-effects (Aung et al., 2017). Furthermore, CHM and the related natural compounds exhibit protective effects against NPC (Kong et al., 2018; Song et al., 2019; Zhao et al., 2019; Guo et al., 2020). Consequently, these are investigated as alternative therapies for use, combined with conventional therapies, to improve the treatment of patients with NPC, particularly advance-stage NPC (Chen et al., 2019).

To evaluate the effect of CHM as a complementary therapy in patients with NPC, particularly advanced-stage NPC, we used a database in Taiwan to explore the effect of CHM on overall mortality. The CHM prescription pattern in NPC with lower overall mortality was also investigated.

## Materials and Methods

### Database Source

This study was performed using the Taiwan Cancer Registry (Long Form) database of the National Health Insurance Research Database (NHIRD) (http://tcr.cph.ntu.edu.tw/main.php?Page=N1) (Chiang et al., 2019). There were detailed TNM stage (TNM (tumor, lymph node, and metastasis), cancer stages and cause of death in this database. This database offered longitudinally linked data for each individual during the period between 2003 and 2016. All personal data were decoded. Informed consent was not required. This study was approved by the Institutional Review Board of the China Medical University Hospital (ethics approval number: CMUH107-REC3-074(CR1)).

### Study Subjects

**FIGURE 1:**
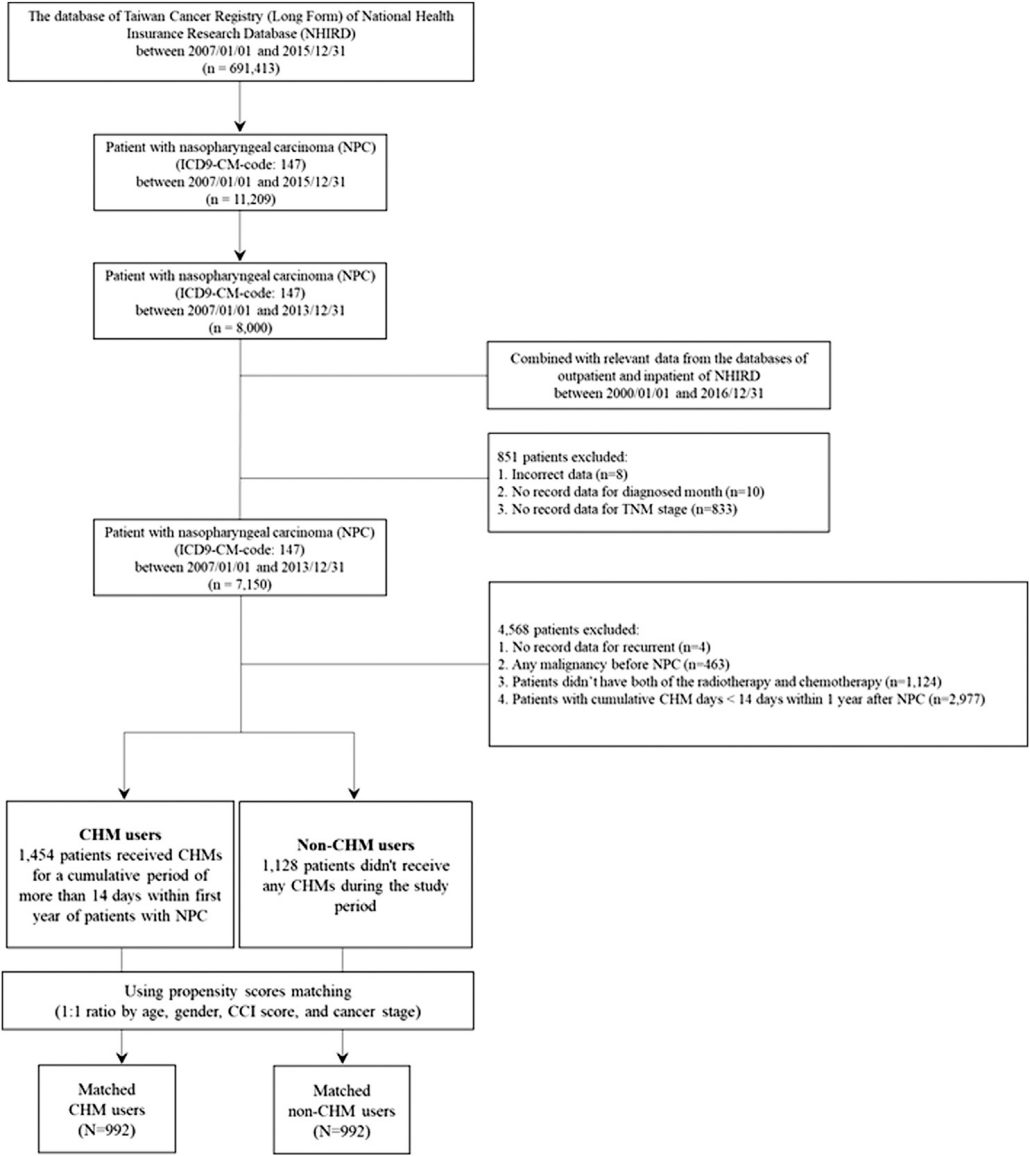
Flowchart of nasopharyngeal carcinoma patient enrollment.

The International Classification of Disease, 9th Revision, Clinical Modification (ICD-9-CM) system was used to identify patients with nasopharyngeal carcinoma (NPC) (ICD-9-CM-code: 147). Overall, 7,150 patients with NPC were identified between 2007–2013 (Figure 1). After excluding patients with incorrect data, missing data, malignancy (ICD9-CM-code: 140–208), no radiotherapy or chemotherapy, and cumulative CHM use of <14 days, 1,454 patients were designated as CHM-users and 1,128 patients as non-users, who did not use CHMs during the follow-up period. To diminish potential bias due to confounders, age, gender, Charlson comorbidity index (CCI) score, and cancer stage CHM-users and non-users were applied to match the two groups for a 1:1 ratio using propensity score matching. After matching, there were 992 matched CHM/non-CHM-user pairs (Figure 1 and Table 1). The date was defined as the index date when 14 cumulative CHM days were completed. The CHM-users continued to use CHMs during the follow-up period. The date of death, the date of withdrawal from the NHIRD database, or the date of the end of follow-up (December 31, 2016) was defined as the study endpoint.

**TABLE 1:** Demographic characteristics of patients with nasopharyngeal carcinoma.

Characteristics	Total subjects			Matched subjects		
	CHM users	Non- users	*P*-value	CHM users	Non- users	*P*-value
	N = 1,454	N = 1,128		N = 992	N = 992	
	N (%)	N (%)		N (%)	N (%)	
Age (years old)			***0.015***			0.945
Age<50	774 (53.23%)	536 (47.52%)		494 (49.80%)	489 (49.29%)	
50≥Age<60	443 (30.47%)	380 (33.69%)		320 (32.26%)	327 (32.96%)	
Age≥60	237 (16.30%)	212 (18.79%)		178 (17.94%)	176 (17.74%)	
Gender			***<0.001***			0.901
Male	1054 (72.49%)	971 (86.08%)		842 (84.88%)	840 (84.68%)	
Female	400 (27.51%)	157 (13.92%)		150 (15.12%)	152 (15.32%)	
CCI score (Mean±SD)	0.87± 1.22	0.67± 1.11	***<0.001***	0.72± 1.06	0.71± 1.13	0.918
T-stage			***<0.001***			1.000
T1-T2	845 (58.12%)	576 (51.06%)		539 (54.33%)	539 (54.33%)	
T3-T4	609 (41.88%)	552 (48.94%)		453 (45.67%)	453 (45.67%)	
N-stage			***<0.001***			0.529
N0	179 (12.31%)	123 (10.90%)		120 (12.10%)	115 (11.59%)	
N1-N2	1072 (73.73%)	766 (67.91%)		706 (71.17%)	692 (69.76%)	
N3-N4	203 (13.96%)	239 (21.19%)		166 (16.73%)	185 (18.65%)	
M-stage			***<0.001***			0.196
M0	1398 (96.15%)	1048 (92.91%)		945 (95.26%)	932 (93.95%)	
M1	56 (3.85%)	80 (7.09%)		47 (4.74%)	60 (6.05%)	
Cancer stage			***<0.001***			0.983
1	47 (3.23%)	31 (2.75%)		32 (3.23%)	31 (3.13%)	
2	392 (26.96%)	231 (20.48%)		229 (23.08%)	223 (22.48%)	
3	568 (39.06%)	381 (33.78%)		359 (36.19%)	366 (36.90%)	
4	447 (30.74%)	485 (43.00%)		372 (37.50%)	372 (37.50%)	
Surgery						
No	1401 (96.35%)	1066 (94.50%)	***0.024***	948 (95.56%)	942 (94.96%)	0.526
Yes	53 (3.65%)	62 (5.50%)		44 (4.44%)	50 (5.04%)	

N, number; CHM, chinese herbal medicine; CCI, charlson comorbidity index; T-stage, tumor stage; N-stage, lymph nodes stage; M-stage, metastasis stage.

Age, gender, TNM stage, cancer stage, and surgery were expressed as categorical variable (number (%)).

Nasopharyngeal carcinoma (ICD-9-CM-code: 147).

*P*-values were obtained by chi-square test. Significant *p-*values (*p* < 0.05) were highlighted in bold italic.

Propensity score matching was performed for age, gender, CCI score, and cancer stage (1:1 ratio).

The Charlson comorbidities include congestive heart failure (ICD-9-CM: 398.91, 402.01, 402.11, 402.91, 404.01, 404.03, 404.11, 404.13, 404.91, 404.93, 425.4 - 425.9, 428.x), peripheral vascular disease (ICD-9-CM: 093.0, 437.3, 440.x, 441.x, 443.1 - 443.9, 447.1, 557.1, 557.9, V43.4), cerebrovascular disease (ICD-9-CM: 362.34, 430.x - 438.x), chronic pulmonary disease (ICD-9-CM: 416.8, 416.9, 490.x - 505.x, 506.4, 508.1, 508.8), rheumatic disease (ICD-9-CM: 446.5, 710.0 - 710.4, 714.0 - 714.2, 714.8, 725.x), peptic ulcer disease (ICD-9-CM: 531.x - 534.x), mild liver disease (ICD-9-CM: 070.22, 070.23, 070.32, 070.33, 070.44, 070.54, 070.6, 070.9, 570.x, 571.x, 573.3, 573.4, 573.8, 573.9, V42.7), diabetes without chronic complication (ICD-9-CM: 250.0 - 250.3, 250.8, 250.9), diabetes with chronic complication (ICD-9-CM: 250.4 - 250.7), hemiplegia or paraplegia (ICD-9-CM: 334.1, 342.x, 343.x, 344.0 - 344.6, 344.9), renal disease (ICD-9-CM: 403.01, 403.11, 403.91, 404.02, 404.03, 404.12, 404.13, 404.92, 404.93, 582.x, 583.0 - 583.7, 585.x, 586.x, 588.0, V42.0, V45.1, V56.x), and any malignancy, including lymphoma and leukemia, except malignant neoplasm of skin (ICD-9-CM: 140.x - 172.x, 174.x - 195.8, 200.x - 208.x, 238.6). These comorbidities were recorded before the diagnosis of nasopharyngeal carcinoma.

### Chinese Herbal Medicine

CHM products used in NPC patients contain two types: single herb and herbal formula (Supplementary Table S1). The herbal formula combines at least two single herbs. A single herb is a part of a plant, such as seeds, fruits, flowers, roots, stems, or leaves. Single herb CHM may also be organs of animals, insects, or minerals. In this study, NPC patients received CHM prescriptions from licensed Chinese medicine doctors, and these CHM prescriptions were produced by pharmaceutical manufacturers following Good Manufacturing Practice in Taiwan (Li et al., 2018; Tsai et al., 2019a; Cheng et al., 2019).

### Association Rule

The CHM prescription profile was investigated using association rule mining (Yang et al., 2013). The association rule was also implemented as previously described for paired CHM combinations (Tsai et al., 2019b; Cheng et al., 2019; Tsai et al., 2019c; Chen et al., 2021; Tsai et al., 2020) using SAS software (version 9.4; SAS Institute, Cary, NC, USA). The strength of association between paired CHM combinations (CHM products X and Y) was shown using the support value (X) (%), confidence value (CHM_X→ CHM_Y; %), and lift value as previously described (Tsai et al., 2019c; Chen et al., 2021; Tsai et al., 2020) (Table 4).

### Network Analysis

Network analysis for CHM clusters was accomplished as previously described (Tsai et al., 2019b; Cheng et al., 2019; Tsai et al., 2019c; Chen et al., 2021; Tsai et al., 2020) using Cytoscape (https://cytoscape.org/, version 3.7.0). The herbal formula is shown as a red circle, and a single herb is expressed as a green circle. The circle size indicates the prescription frequency of the CHM. The line size signifies the support value between paired CHM products. Line color displays the lift value between paired CHM products. The thicker and darker connection line shows a stronger connection strength between the paired CHM products.

### Statistical Analysis

Categorical data (age, gender, TNM [tumor, lymph node, and metastasis] stage, cancer stage, and surgery) are shown as numbers (percentages), and the Chi-squared test was applied to evaluate the differences between CHM-users and non-users (Table 1). Crude and adjusted Cox proportional hazard models were used to estimate the risk of overall mortality (Table 2). The adjustment factors included age, gender, CHM use, CCI score, cancer stage, and surgery (Table 2). NPC patients were stratified according to cancer stage (Table 3 and Figure 4). For NPC patients in cancer stages 1 + 2, patients were stratified by age, gender, and CCI (Table 3 and Figure 4). For NPC patients in cancer stages 3 + 4, patients were also stratified by age, gender, and CCI (Table 3 and Figure 4). The adjustment factors included age, gender, CHM use, and CCI score (Table 3). Kaplan‒Meier curves and log-rank tests were performed to assess the cumulative incidence of overall mortality between the two groups (Figure 3). *p*-values of less than 0.05 were considered statistically significant. All analyses were completed using SAS software (version 9.4; SAS Institute).

**TABLE 2:** Cox proportional hazard models for overall mortality in patients with nasopharyngeal carcinoma in Taiwan.

	Crude			Adjusted		
	HR	(95% CI)	*P*-value	aHR	(95% CI)	*P*-value
Age (years old)						
Age<50	Ref.	ND	ND	Ref.	ND	ND
50≥Age<60	1.17	(0.97–1.43)	0.1067	1.20	(0.98–1.46)	0.0777
Age≥60	2.37	(1.95–2.89)	***<.0001***	2.36	(1.9–2.93)	***<.0001***
Gender						
Male	Ref.	ND	ND	Ref.	ND	ND
Female	0.77	(0.6–0.98)	***0.0360***	0.73	(0.57–0.95)	***0.0173***
CHM use						
No	Ref.	ND	ND	Ref.	ND	ND
Yes	0.82	(0.71–0.96)	***0.0125***	0.78	(0.67–0.92)	***0.0024***
CCI score (Mean±SD), per score	1.21	(1.13–1.29)	***<.0001***	1.07	(1–1.16)	0.0553
Cancer stage						
1	Ref.	ND	ND	Ref.	ND	ND
2	1.732	(0.76–3.94)	0.1899	1.709	(0.77–3.78)	0.1865
3	2.869	(1.29–6.39)	***0.0099***	2.818	(1.3–6.12)	***0.0088***
4	6.75	(3.07–14.86)	***<.0001***	6.857	(3.19–14.73)	***<.0001***
Surgery						
No	Ref.	ND	ND	Ref.	ND	ND
Yes	1.12	(0.79–1.58)	0.5239	1.00	(0.69–1.43)	0.9811

CHM, chinese herbal medicine; HR, hazard ratio; 95% CI, 95% confidence interval; ND, not determined; CCI, charlson comorbidity index.

Nasopharyngeal carcinoma (ICD-9-CM-code: 147).

CCI score (Mean±SD) was expressed as a continuous variable. The risk of overall mortality increased with CCI score(HR 1.07/score) in our study.

Models adjusted for age, gender, CHM use, CCI score, cancer stage, and surgery.

*P*-value (*p* < 0.05) was shown in bold italic font.

**TABLE 3:** Subgroup analysis for the risk of overall mortality in patients with nasopharyngeal carcinoma when stratified by cancer stages.

Subgroup	CHM users N=992		Non- users N=992		Crude			Adjusted		
	Event	All	Event	All	HR	(95%CI)	*P*-value	aHR	(95%CI)	*P*-value
Cancer stages (all)										
Overall	280	992	323	992	0.822	(0.705–0.959)	***0.0125***	0.813	(0.697–0.947)	***0.0081***
Age (Mean±SD)	280	992	323	992	1.035	(1.027–1.043)	***<.0001***	1.032	(1.024–1.040)	***<.0001***
Gender										
Male	249	842	278	840	Ref.	ND	ND	Ref.	ND	ND
Female	31	150	45	152	0.766	(0.597–0.983)	***0.036***	0.768	(0.598–0.985)	***0.0379***
CCI (Mean±SD)	280	992	323	992	1.206	(1.128–1.290)	***<.0001***	1.099	(1.022–1.181)	***0.0105***
										
Cancer stages 1+2										
Overall	35	261	42	254	0.791	(0.506–1.236)	0.3031	0.84	(0.534–1.323)	0.4527
Age (Mean±SD)	35	261	42	254	1.035	(1.008–1.062)	***0.0116***	1.027	(1.000–1.054)	***0.0481***
Gender										
Male	30	222	35	207	Ref.	ND	ND	Ref.	ND	ND
Female	5	39	7	47	0.86	(0.438–1.688)	0.6612	0.816	(0.419–1.589)	0.549
CCI (Mean±SD)	35	261	42	254	1.369	(1.155–1.622)	***0.0003***	1.277	(1.084–1.503)	***0.0034***
Cancer stages 3+4										
Overall	245	731	281	738	0.825	(0.699–0.973)	***0.0223***	0.799	(0.676–0.943)	***0.008***
Age (Mean±SD)	245	731	281	738	1.036	(1.028–1.044)	***<.0001***	1.034	(1.026–1.042)	***<.0001***
Gender										
Male	219	620	243	633	Ref.	ND	ND	Ref.	ND	ND
Female	26	111	38	105	0.771	(0.587–1.011)	0.0603	0.791	(0.604–1.036)	0.0891
CCI (Mean±SD)	245	731	281	738	1.172	(1.094–1.255)	***<.0001***	1.057	(0.978–1.141)	0.1597

CHM, Chinese herbal medicine; HR, hazard ratio; aHR, adjusted hazard ratio; 95% CI, 95% confidence interval; ND, not determined; CCI, Charlson comorbidity index. Nasopharyngeal carcinoma (ICD-9-CM-code: 147).

Models adjusted for age, gender, and CCI score. *P*-value (*p* < 0.05) was shown in bold italic font.

**TABLE 4:** Ten most commonly used pairs of CHM products for patients with nasopharyngeal carcinoma in Taiwan.

CHM products (LHS, X)	Chinese name	Frequency of prescriptions of X product		CHM products (RHS, Y)	Chinese name	Frequency of prescriptions of Y product	Frequency of prescriptions of X and Y products	Support (X) (%)	Confidence (X →Y) (%)	Lift
Ban-Zhi-Lian (BZL)	半枝蓮	2040	→	Bai-Hua-She-She-Cao (BHSSC)	白花蛇舌草	5719	1596	4.9	78.2	4.5
Sheng-Di-Huang (ShengDH)	生地黃	2232	→	Mai-Men-Dong (MMD)	麥門冬	3140	1041	3.2	46.6	4.9
Gan-Lu-Yin (GLY)	甘露飲	5185	→	Bai-Hua-She-She-Cao (BHSSC)	白花蛇舌草	5719	998	3.0	19.2	1.1
Mai-Men-Dong (MMD)	麥門冬	3140	→	Xuan-Shen (XS)	玄參	3323	930	2.8	29.6	2.9
Sheng-Di-Huang (ShengDH)	生地黃	2232	→	Xuan-Shen (XS)	玄參	3323	876	2.7	39.2	3.9
Xin-Yi-Qing-Fei-Tang (XYQFT)	辛夷清肺湯	3812	→	Bai-Hua-She-She-Cao (BHSSC)	白花蛇舌草	5719	823	2.5	21.6	1.2
Gua-Lou-Gen (GLG)	栝樓根	3156	→	Bai-Hua-She-She-Cao (BHSSC)	白花蛇舌草	5719	781	2.4	24.7	1.4
Gua-Lou-Gen (GLG)	栝樓根	3156	→	Gan-Lu-Yin (GLY)	甘露飲	5185	747	2.3	23.7	1.5
Mai-Men-Dong (MMD)	麥門冬	3140	→	Bai-Hua-She-She-Cao (BHSSC)	白花蛇舌草	5719	735	2.2	23.4	1.3
Mai-Men-Dong (MMD)	麥門冬	3140	→	Gua-Lou-Gen (GLG)	栝樓根	3156	729	2.2	23.2	2.4

CHM, Chinese herbal medicine; LHS, left-hand-side; RHS, right-hand-side.

Total prescriptions = 32842.

Support (X) (%) = Frequency of prescription of X and Y products / total prescriptions x 100%.

Confidence (X →Y) (%) = Frequency of prescription of X and Y products / Frequency of prescription of X product x 100%.

P (Y) (%) = Frequency of prescription of Y product / total prescriptions x 100%.

Lift = Confidence (X →Y) (%) / P (Y) (%).

## Results

### Demographic Characteristics

For CHM-users and non-users, age, gender, CCI score, TNM stage, cancer stage, and surgery differed significantly (total subjects; *p-*value < 0.05; Table 1). To decrease confounding effects, propensity score matching was performed, after which there were no significant differences in demographic characteristics between the two matched users (*p-*value > 0.05).

### Overall Mortality

In the investigation of overall mortality in patients with NPC (Table 2), the crude Cox proportional hazard model revealed significant differences in age, gender, CHM use, CCI score, and cancer stage. After adjusting for these variances, the adjusted Cox proportional hazard model showed that patients aged over 60 years had a higher overall mortality risk than those aged below 50 years (Table 2; adjusted hazard ratio [aHR]: 2.36, 95% CI: 1.90–2.93, *p* < 0.0001). Females showed a lower risk of overall mortality than males (Table 2; aHR: 0.73, 95% CI: 0.57–0.95, *p* = 0.0173). Patients with cancer stage 3 had a higher risk of overall mortality than those with cancer stage 1 (Table 2; aHR: 2.82, 95% CI: 1.30‒6.12, *p* = 0.0088). Patients with cancer stage 4 had a higher risk of overall mortality than those with cancer stage 1 (Table 2; aHR: 6.86, 95% CI: 3.19‒14.73, *p* < 0.0001).

**FIGURE 2:**
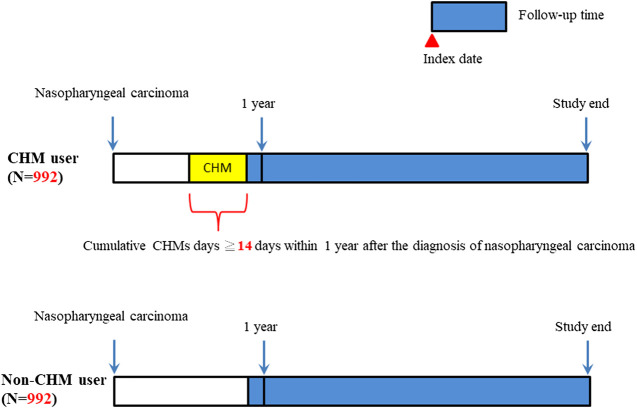
Diagram of follow-up time for NPC patients. Abbreviations: NPC, nasopharyngeal carcinoma.

CHM-users showed a lower overall mortality risk than non-users (aHR: 0.78, 95% CI: 0.67–0.92, *p* = 0.0024; Table 2). Kaplan‒Meier survival plots revealed the difference in the cumulative incidence of overall mortality between the two groups of users (Figure 2; *p* < 0.0001, log-rank test). The cumulative incidence of overall mortality was significantly higher in non-users.

**FIGURE 3:**
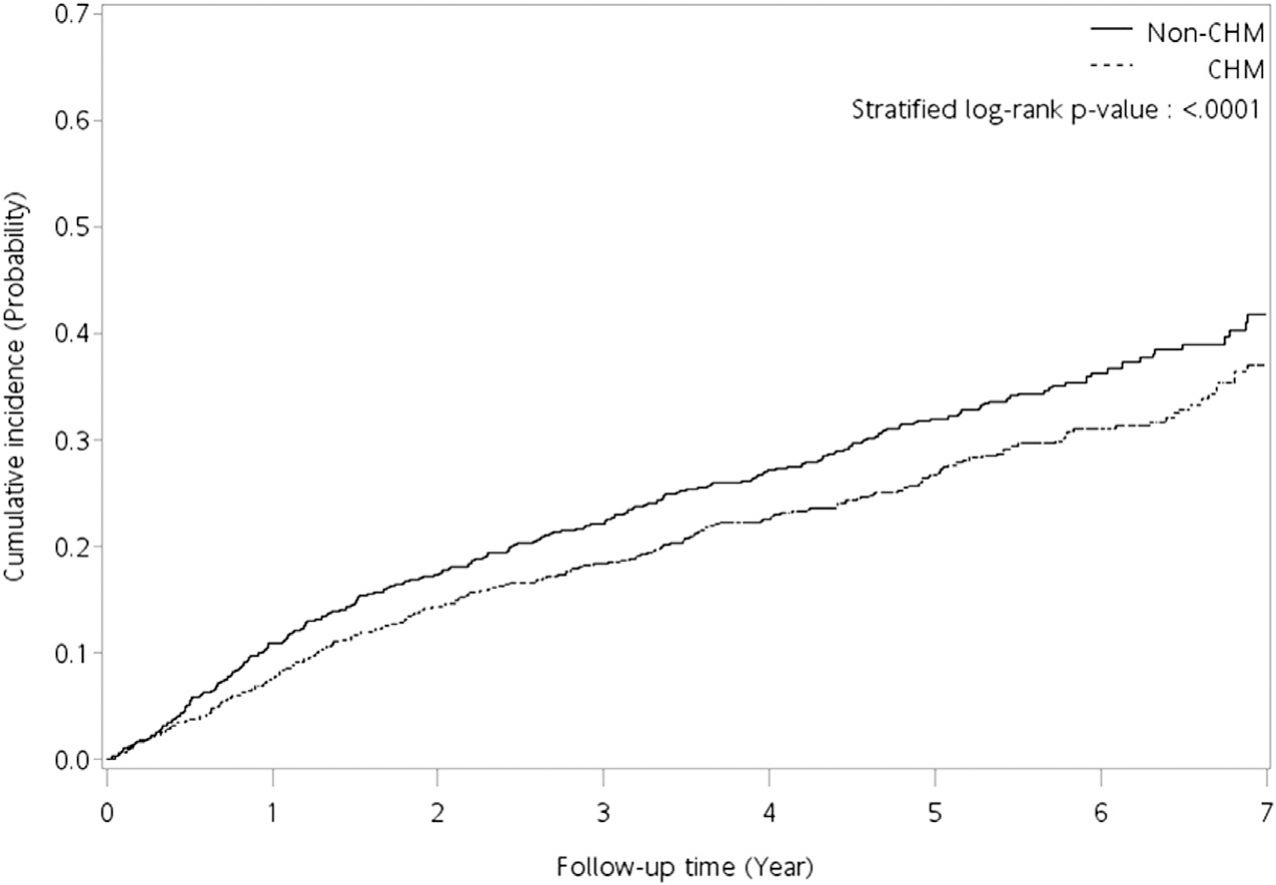
Kaplan‒Meier curves for overall mortality for NPC patients. Abbreviations: CHM, chinese herbal medicine; NPC, nasopharyngeal carcinoma.

**FIGURE 4:**
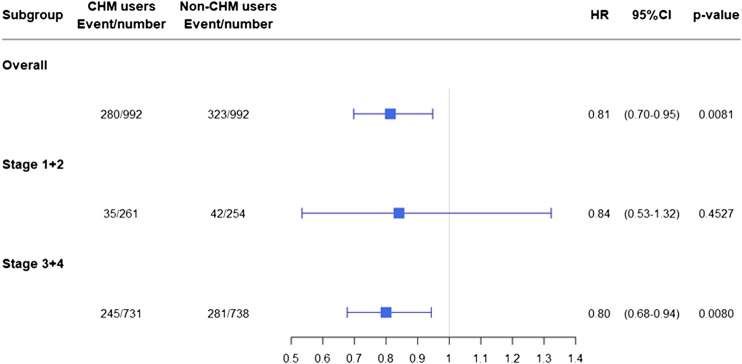
Risk of overall mortality in NPC patients when stratified by cancer stages. Abbreviations: NPC, nasopharyngeal carcinoma; CHM, chinese herbal medicine; HR, hazard ratio; CI, confidence interval.

The hazard ratios for overall mortality in these NPC patients were separated into subgroups according to cancer stage. Among these subgroups, a lower overall mortality risk was observed in CHM-users in patients with cancer stages 3 + 4 (advanced stages; aHR: 0.799, 95% CI: 0.676–0.943, *p* = 0.008) (Table 3 and Figure 4).

### Chinese Herbal Medicine Prescription Pattern

The herbal composition and related prescription frequency information for patients with NPC are listed in Table S1. Based on prescription frequency, Gan-Lu-Yin (GLY) was the most commonly prescribed herbal formula, Xin-Yi-Qing-Fei-Tang (XYQFT) was the second herbal formula. For single herbs, Bai-Hua-She-She-Cao (*Herba Hedyotis Diffusae*; *Hedyotis diffusa Willd.*) was most prescribed single herb, followed by Xuan-Shen (*Radix Scrophulariae*; *Scrophularia ningpoensis Hensl.*) and Gua-Lou-Gen (*Radix Trichosanthis*; *Trichosanthes kirilowii Maxim.*).

Association rule analysis revealed the CHM product pairs most used for patients with NPC (Table 4). Higher levels of support, confidence, and lift indicated stronger associations for paired CHM products. The most commonly used paired CHM products were Ban-Zhi-Lian (BZL)→Bai-Hua-She-She-Cao (BHSSC) (first co-prescription frequency: 1,596, support: 4.9%, confidence: 78.2%, lift: 4.5), followed by Sheng-Di-Huang (ShengDH)→Mai-Men-Dong (MMD) (second co-prescription frequency: 1,041, support: 3.2%, confidence: 46.6%, lift: 4.9), and GLY→BHSSC (third co-prescription frequency: 998, support: 3.0%, confidence: 19.2%, lift: 1.1) (Table 4).

**FIGURE 5:**
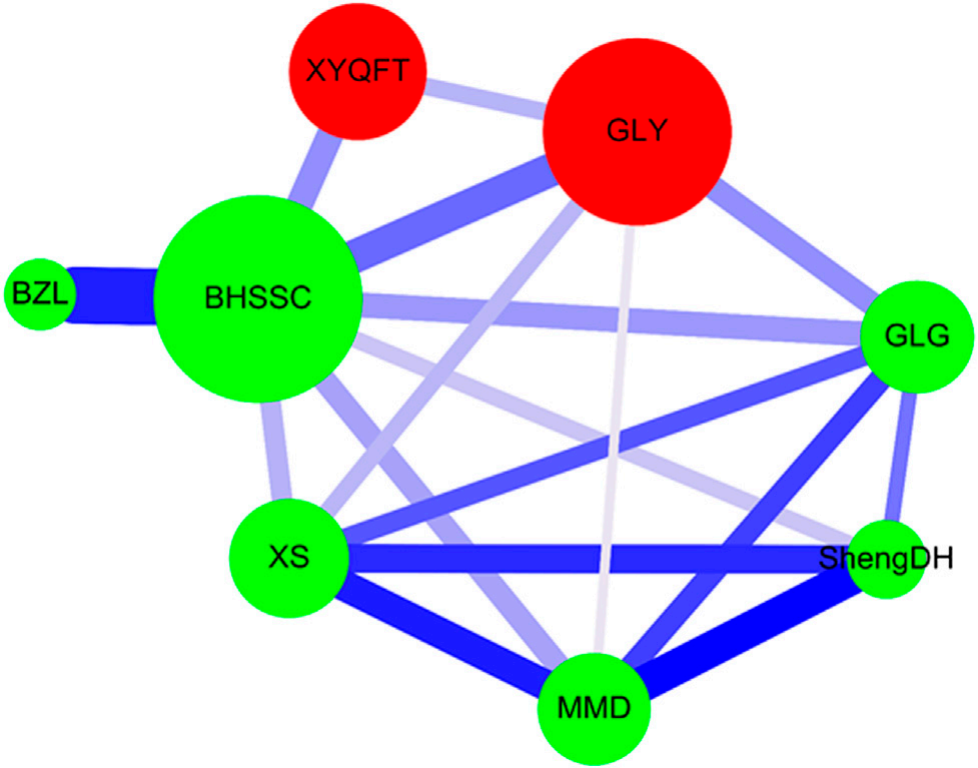
CHM network analysis in NPC patients. Herbal formula is shown as the red circle, and single herb is expressed as the green circle. The circle size indicates prescription frequency of the CHM. The line size and color signify the support value and the lift value between paired CHM products, respectively. The thicker and darker connection line shows the stronger strength of connection between the paired CHM products. Abbreviations: NPC, nasopharyngeal carcinoma; CHM, chinese herbal medicine.

Network analysis revealed the CHM prescription network for patients with NPC (Figure 5). There were 992 patients who used 32,842 prescriptions by traditional Chinese medicine doctors (Table 4). Network analysis revealed two clusters (Figure 5). In cluster 1, BHSSC showed the core CHM. BZL, GLY, and XYQFT were nearby CHMs. In cluster 2, ShengDH, MMD, XS, and GLG were important CHMs. Our results show that these 8 CHMs are important for patients with NPC.

## Discussion

The long-term therapeutic effects of CHM in patients with NPC, particularly advanced-stage NPC, remain to be elucidated (Kim et al., 2015; Song et al., 2019). In our study, with the TNM and cancer stage information and NPC patients identified from the database of the Taiwan Cancer Registry (Long Form) of the NHIRD, we were able to assess the CHM effects for long-term use in NPC patients with advanced-stage disease (cancer stages 3 + 4). NPC patients who used CHM had lower overall and cancer-related mortality than those who did not use CHM after a 7-years follow-up. There was 88.2% of NPC patients who had died from the various forms of cancer (malignancies; ICD9-CM-code: 140–208; Supplementary Figure S2). There were 81.3% of NPC patients who had died from NPC cancer (ICD9-CM-code: 147; Supplementary Figure S2). Only 6.9% of NPC patients who had died from other than NPC cancer (malignancies; ICD9-CM-code: 140–208, except for 147; Supplementary Figure S2). There was only 1.5% of NPC patients who had died from cardiocerebrovascular diseases (ICD9-CM-code: 390–459; Supplementary Figure S2). For NPC patients, CHM-users showed a lower cancer-related mortality risk than non-users (Supplementarys Table S9; Figure S6). Also, the NPC-related mortality risk was lower for CHM users after controlling for age, gender, and CCI score for these NPC patients (Supplementarys Table S10; Figure S7).

For advanced NPC patients, the overall mortality risk was 0.799-fold (95%CI: 0.676–0.943) for CHM-users after controlling for age, gender, and CCI score. The dose and duration of using CHM was associated with a reduced risk of overall mortality among patients with NPC (Supplementarys Tables S3–S8; Figures S1–S3). There was 88.6% of advanced NPC patients who had died from the various forms of cancer (malignancies; ICD9-CM-code: 140–208; Supplementary Figure S5). There were 82.9% of advanced NPC patients who had died from NPC cancer (ICD9-CM-code: 147; Supplementary Figure S5). Only 5.7% of advanced NPC patients who had died from other than NPC cancer (malignancies; ICD9-CM-code: 140–208, except for 147; Supplementary Figure S5). There was only 1.7% of advanced NPC patients who had died from cardiocerebrovascular diseases (ICD9-CM-code: 390–459; Supplementary Figure S5). For advanced NPC patients, CHM-users showed a lower cancer-related mortality risk than non-users (Supplementarys Table S11; Figure S8). Similar result was also observed in the NPC-related mortality. The NPC-related mortality risk was lower for CHM users after controlling for age, gender, and CCI score for these advanced NPC patients (Supplementarys Table S13; Figure S9). Furthermore, we found that eight CHMs were important for these advanced NPC patients by association rules and network analyses. These results provide the utility of clinical CHM as a complementary therapy for patients with advanced NPC.

We enrolled primary NPC patients who received both radiotherapy and chemotherapy. Approximately 80% of these patients were <60 years old and about 80% were male. Our results are similar to those of previous studies (Song et al., 2019). NPC patients in Taiwan were characterized by more males, and 80% of them were under 60 years old in another study (Song et al., 2019). The 5-years overall mortality for NPC patients was approximately 30% in our study, which is in agreement with previous studies (Ji et al., 2019; Zhu et al., 2019). We found that the 5-years overall mortality for patients with NPC was about 25% when patients used CHM. Several Chinese herbs and compounds exhibit protective effects against NPC (Kong et al., 2018; Song et al., 2019; Zhao et al., 2019; Guo et al., 2020). Our results showed the protective effects of the clinical use of CHMs against overall mortality in NPC patients, particularly those with advanced stages.

Our association rule analysis showed that the most commonly used CHM pairs were BZL →BHSSC, followed by ShengDH→MMD, and GLY→BHSSC. Our network analysis showed that, in cluster 1, BHSSC showed the core CHM, and BZL, GLY, and XYQFT were nearby CHMs. In cluster 2, ShengDH, MMD, XS, and GLG were important CHMs.

We identified single herbs, including BZL, BHSSC, ShengDH, MMD, XS, and GLG. Among these single herbs, BZL and BHSSC show anti-cancer and anti-inflammatory activities (Perez et al., 2010; Zhang et al., 2017). BZL is the entire plant of *Scutellaria barbata D. Don* (the *Lamiaceae* family). Interestingly, natural compounds of BZL, including scutebarbatines, barbatin D, barbatin E, 3′,4′,5,7-tetrahydroxyflavone, 5,7,4′-trihydroxyflavone (apigenin), and quercetin show significant cytotoxic activities against NPC cells (Ong et al., 2004; Dai et al., 2008; Dai et al., 2011; Daker et al., 2012; Li et al., 2014; Wu et al., 2017). Notably, apigenin also inhibits Epstein‒Barr virus (EBV) reactivation (Wu et al., 2017). Scleromitrion diffusum (Willd.) R.J.Wang (syn. Hedyotis diffusa Willd. (Rubiaceae family)). Natural compounds of BHSSC, including shecaocerenoside A, shecaoiridoidside C, coumarin, and quercetin, exhibit anti-tumor activity, including human NPC cells (Wang et al., 2017; Peng et al., 2018).

GLY contains ten single herbs. GLY has shown anti-NPC activity in NPC patients (Song et al., 2019). The GLY extract also shows anti-angiogenic effects (Pan et al., 2010). Our advanced NPC patients also used GLY, with a better survival rate. The natural compound epigallocatechin-3-gallate (EGCG) is from GLY and inhibits human NPC cell migration by suppressing MMP-2 expression (Ho et al., 2019). EGCG also suppresses NPC cell growth by attenuating STAT3 activation (Lin et al., 2014). 5,7-dihydroxyflavone promotes human NPC cell apoptosis via tumor necrosis factor-related apoptosis-inducing ligands (Li et al., 2011). Genistein induces NPC cell growth inhibition and G2/M arrest (Han et al., 2010). Genistein also suppresses NPC stem cell growth via sonic hedgehog signaling (Zhang et al., 2019). Other natural compounds of GLY, including apigenin, coumarin, and quercetin show significant cytotoxic activities against NPC cells (Ong et al., 2004; Daker et al., 2012; Peng et al., 2018).

XYQFT contains 10 single herbs and is prescribed to treat respiratory-related diseases, including asthma and allergic rhinitis (Yen et al., 2015; Lo et al., 2020). XYQFT also contains natural compounds, including EGCG, apigenin, coumarin, genistein, and quercetin, which exhibit anti-NPC activities (Ong et al., 2004; Han et al., 2010; Daker et al., 2012; Lin et al., 2014; Peng et al., 2018; Ho et al., 2019; Zhang et al., 2019). Furthermore, 1,3,8-trihydroxy-6-methylanthraquinone (emodin) from XYQFT inhibits EBV reactivation and suppresses NPC cell proliferation (Ma et al., 2017; Wu et al., 2019). The 3-phenyl-2-propenal (*trans*-cinnamaldehyde) inhibits NPC cells (Daker et al., 2013).

This study demonstrated that complementary CHM therapy may reduce overall and cancer related mortality among advanced NPC patients. There are eight clinically used CHM products that are potentially useful for advanced NPC patients. However, the actual dose of specific CHMs in this prescription for patients was unknown, and the metabolism of the co-prescription pattern in humans, as well as potential confounders (i.e., body mass index, fatty tissue, lifestyle, personalized treatments, social-economic status, and cigarette smoking etc.), were not clarified in this study. Therefore, further randomized controlled trials and functional investigations of these potentially useful CHM products are necessary to validate their efficacy and safety in these patients.

## Data Availability

The raw data supporting the conclusions of this article will be made available by the authors, without undue reservation.
