# Supplementary material for: Effect of Chinese Herbal Medicine Therapy on Overall and Cancer Related Mortality in Patients With Advanced Nasopharyngeal Carcinoma in Taiwan
Source: Front Pharmacol. 2021 Jan 29;11:607413. doi: 10.3389/fphar.2020.607413 (PMC7941275; doi:10.3389/fphar.2020.607413)
Supplement: Supplementary file 1 [file datasheet1.docx]

**Supplementary Information**

Supplementary Text

Figures S1-S9

Table S1-S12

**Supplementary Text**

**Figure S1.** Cumulative incidence of overall mortality between CHM and non-CHM users among patients with nasopharyngeal carcinoma (NPC) (Cumulative days of CHM treatment ≧28 days within the first year of NPC) in Taiwan.

**Figure S2.** Cumulative incidence of overall mortality between CHM and non-CHM users among patients with nasopharyngeal carcinoma (NPC) (Cumulative days of CHM treatment ≧56 days within the first year of NPC) in Taiwan.

**Figure S3.** Cumulative incidence of overall mortality between CHM and non-CHM users among patients with nasopharyngeal carcinoma (NPC) (Cumulative days of CHM treatment ≧84 days within the first year of NPC) in Taiwan.

**Figure S4.** Proportional distribution of causes of death among patients with nasopharyngeal carcinoma (NPC) from 1 January 2007 through 31 December 2015.

**Figure S5.** Proportional distribution of causes of death among patients with advanced nasopharyngeal carcinoma (NPC) (stages 3+4) from 1 January 2007 through 31 December 2015.

**Figure S6.** Cumulative incidence of cancer-related mortality between CHM and non-CHM users among patients with nasopharyngeal carcinoma (NPC) (Cumulative days of CHM treatment ≧14 days within the first year of NPC) in Taiwan.

**Figure S7.** Cumulative incidence of NPC-related mortality between CHM and non-CHM users among patients with nasopharyngeal carcinoma (NPC) (Cumulative days of CHM treatment ≧14 days within the first year of NPC) in Taiwan.

**Figure S8.** Cumulative incidence of cancer-related mortality between CHM and non-CHM users among patients with advanced nasopharyngeal carcinoma (NPC) (stages 3+4) (Cumulative days of CHM treatment ≧14 days within the first year of advanced NPC) in Taiwan.

**Figure S9.** Cumulative incidence of NPC-related mortality between CHM and non-CHM users among patients with advanced nasopharyngeal carcinoma (NPC) (stages 3+4) (Cumulative days of CHM treatment ≧14 days within the first year of advanced NPC) in Taiwan.

**Table S1.** Composition of herbal formulas and single herbs for patients with nasopharyngeal carcinoma in Taiwan.

**Table S2**. Distribution of the cumulative days of CHM treatment during the study period among patients with advanced nasopharyngeal carcinoma.

**Table S3.** Demographic characteristics of patients with nasopharyngeal carcinoma (NPC) (cumulative days of CHM treatment ≧28 days within the first year of NPC).

**Table S4.** Cox proportional hazard models for overall mortality in patients with nasopharyngeal carcinoma (NPC) in Taiwan (cumulative days of CHM treatment ≧28 days within the first year of NPC).

**Table S5.** Demographic characteristics of patients with nasopharyngeal carcinoma (NPC) (cumulative days of CHM treatment ≧56 days within the first year of NPC).

**Table S6.** Cox proportional hazard models for overall mortality in patients with nasopharyngeal carcinoma (NPC) in Taiwan (cumulative days of CHM treatment ≧56 days within the first year of NPC).

**Table S7.** Demographic characteristics of patients with nasopharyngeal carcinoma (NPC) (cumulative days of CHM treatment ≧84 days within the first year of NPC).

**Table S8.** Cox proportional hazard models for overall mortality in patients with nasopharyngeal carcinoma (NPC) in Taiwan (cumulative days of CHM treatment ≧84 days within the first year of NPC).

**Table S9.** Cox proportional hazard models for cancer-related mortality in patients with nasopharyngeal carcinoma (NPC) in Taiwan (cumulative days of CHM treatment ≧14 days within the first year of NPC).

**Table S10.** Cox proportional hazard models for NPC-related mortality in patients with nasopharyngeal carcinoma (NPC) in Taiwan (cumulative days of CHM treatment ≧14 days within the first year of NPC).

**Table S11.** Cox proportional hazard models for cancer-related mortality in patients with advanced nasopharyngeal carcinoma (NPC) (stages 3+4) in Taiwan (cumulative days of CHM treatment ≧14 days within the first year of advanced NPC).

**Table S12.** Cox proportional hazard models for NPC-related mortality in patients with advanced nasopharyngeal carcinoma (NPC) (stages 3+4) in Taiwan (cumulative days of CHM treatment ≧14 days within the first year of advanced NPC).

**Fig. S1**

**
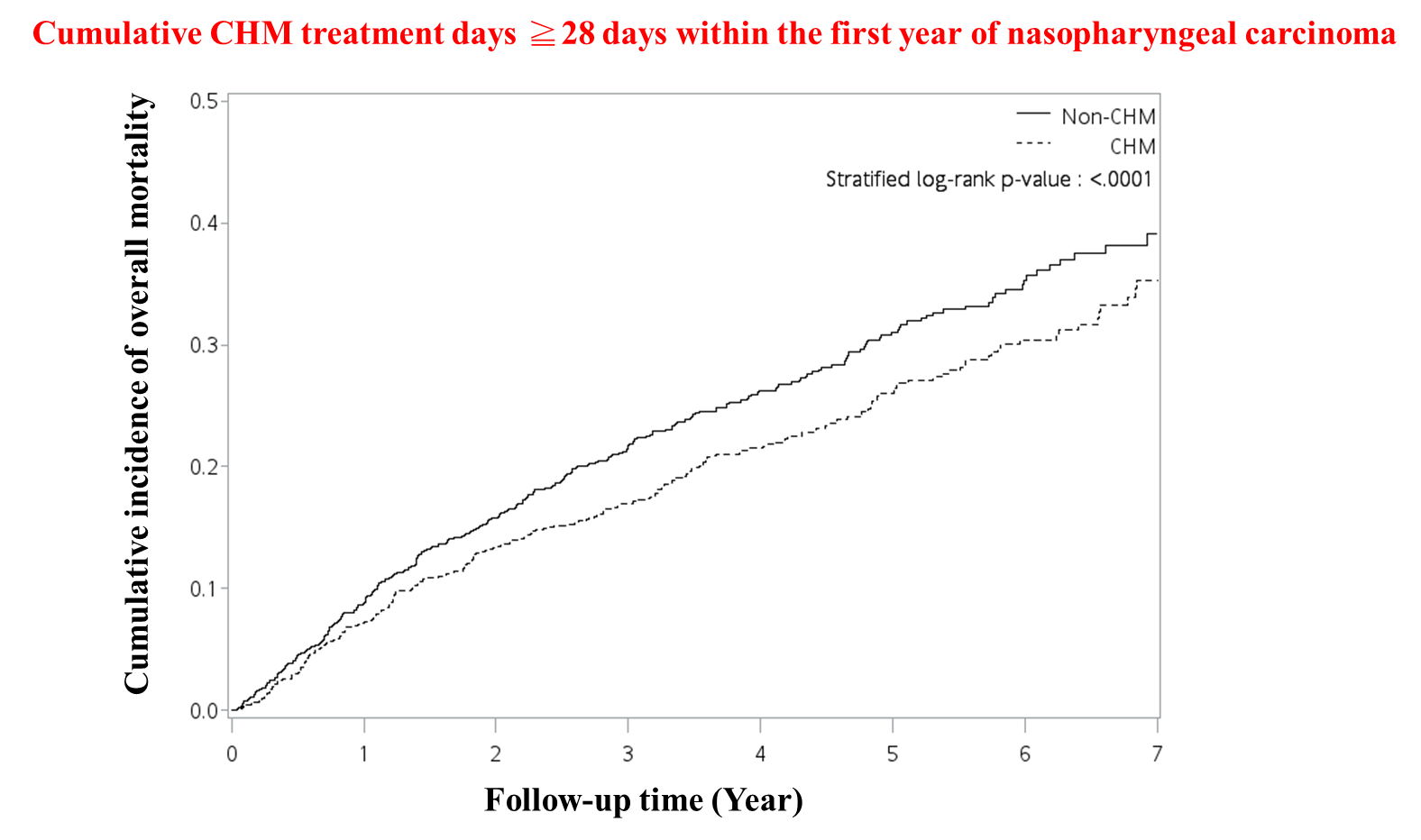
**

**Fig. S2**


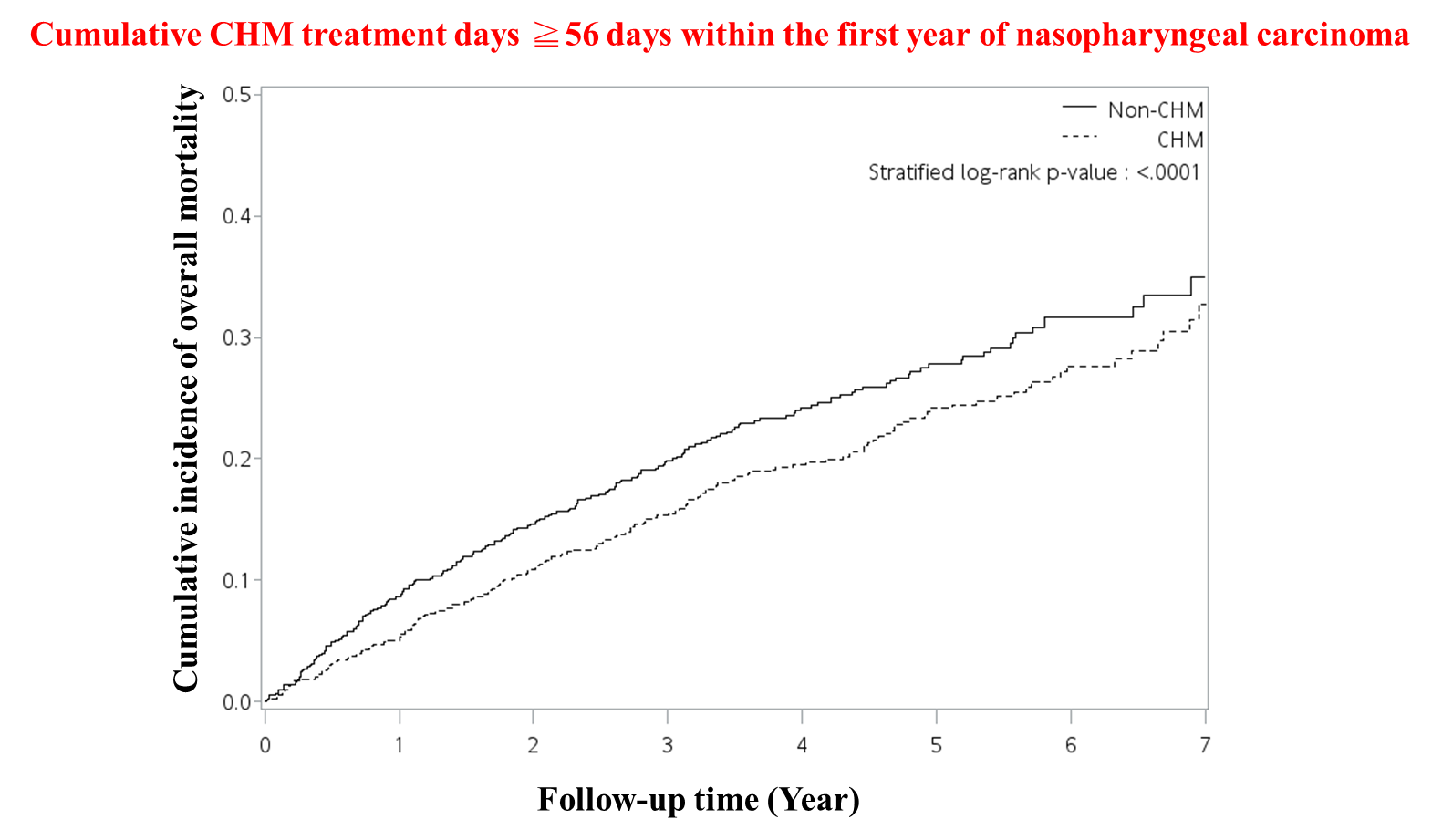


**Fig. S3**


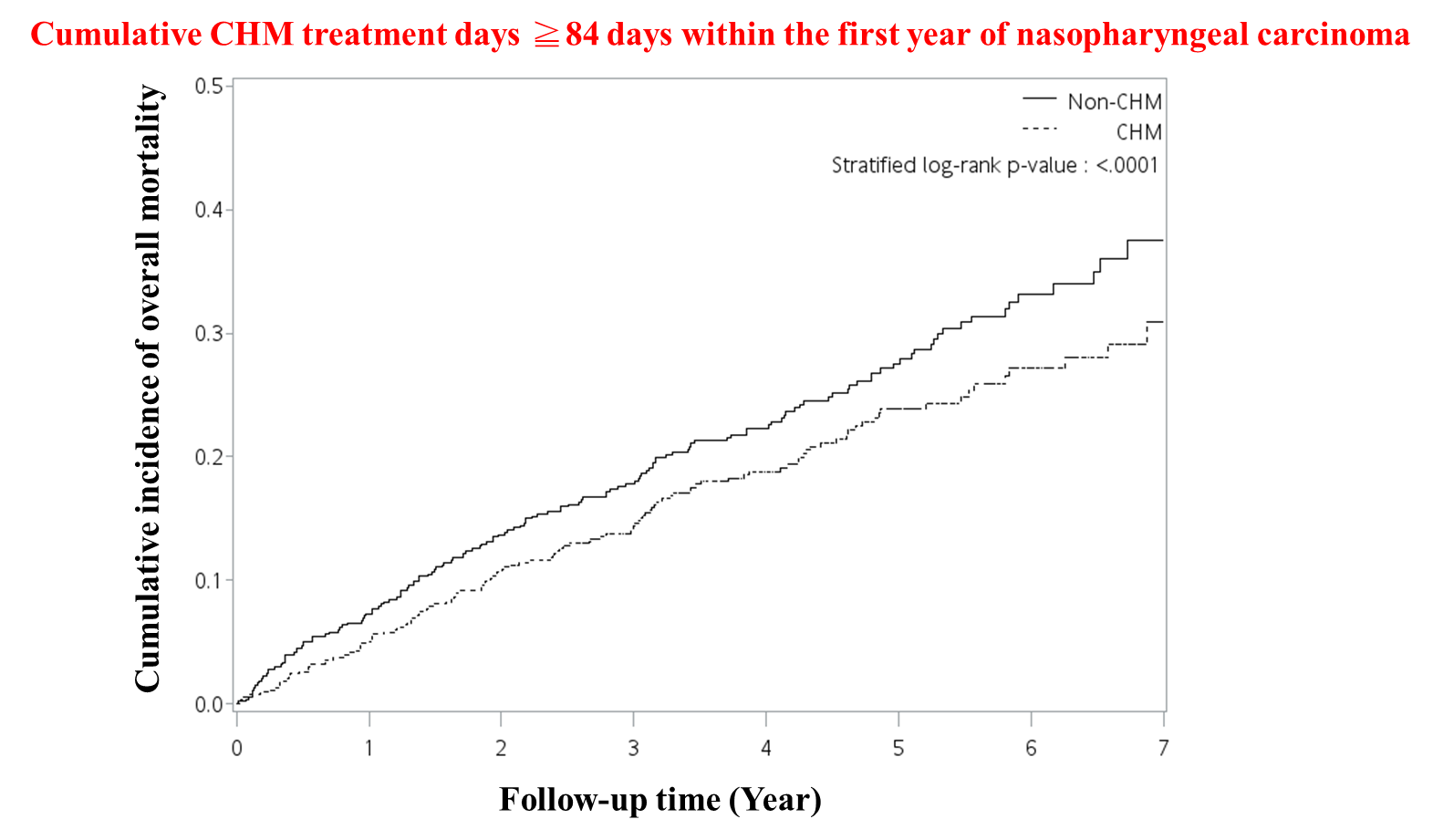


**Fig. S4**


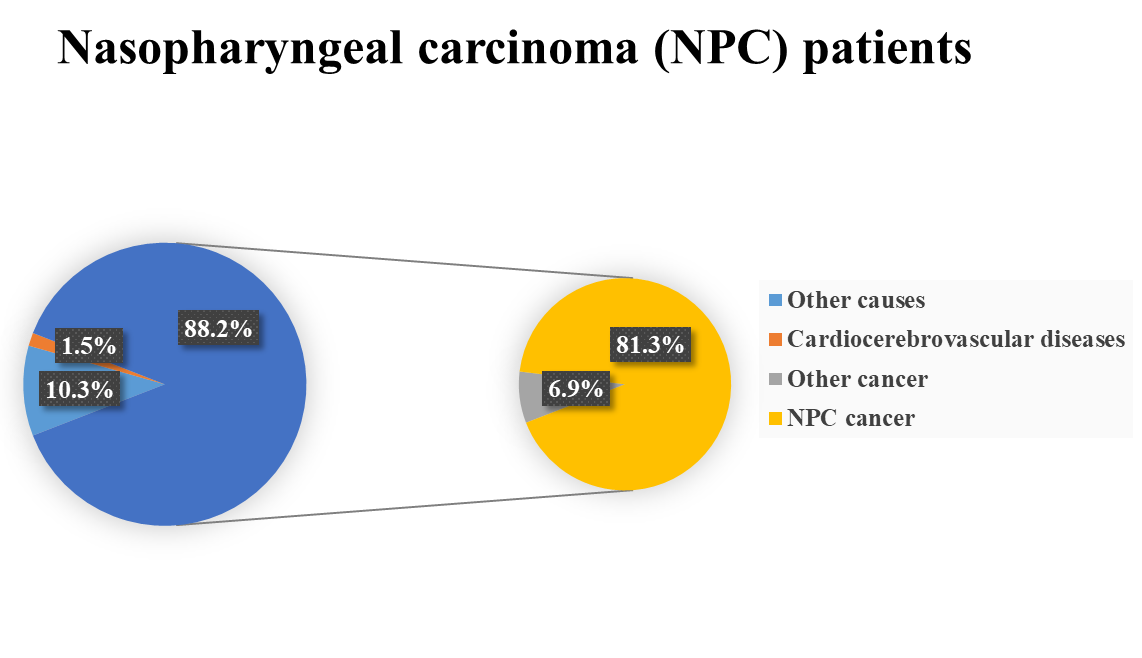


**Fig. S5**


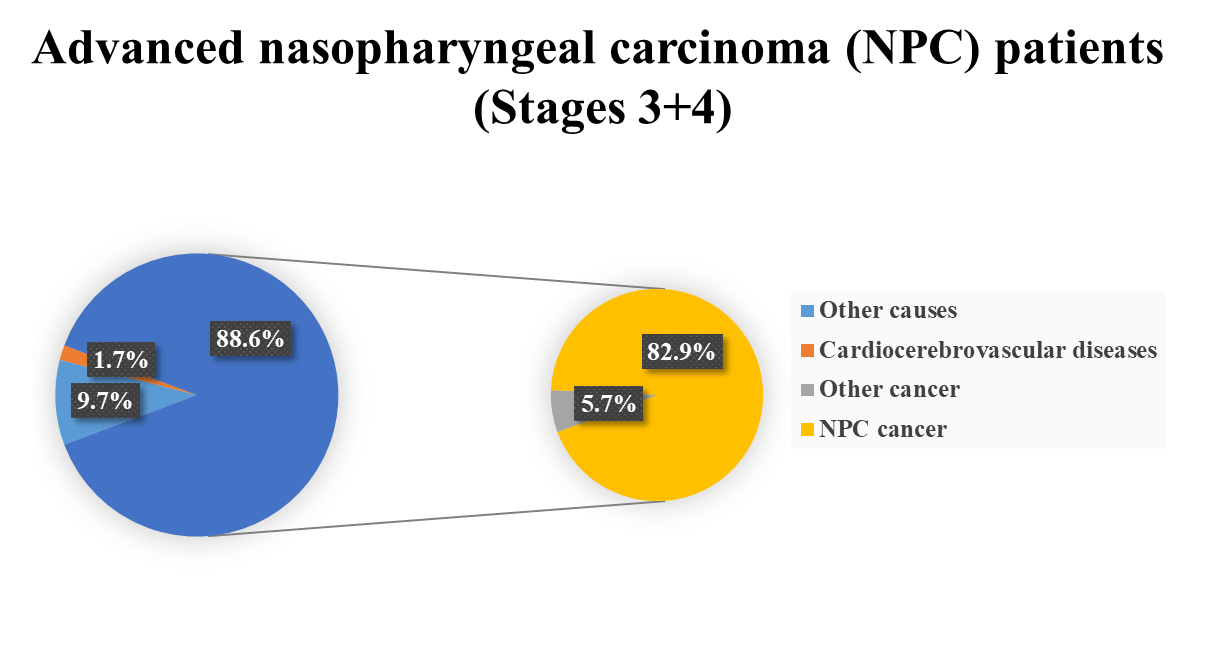


**Fig. S6**


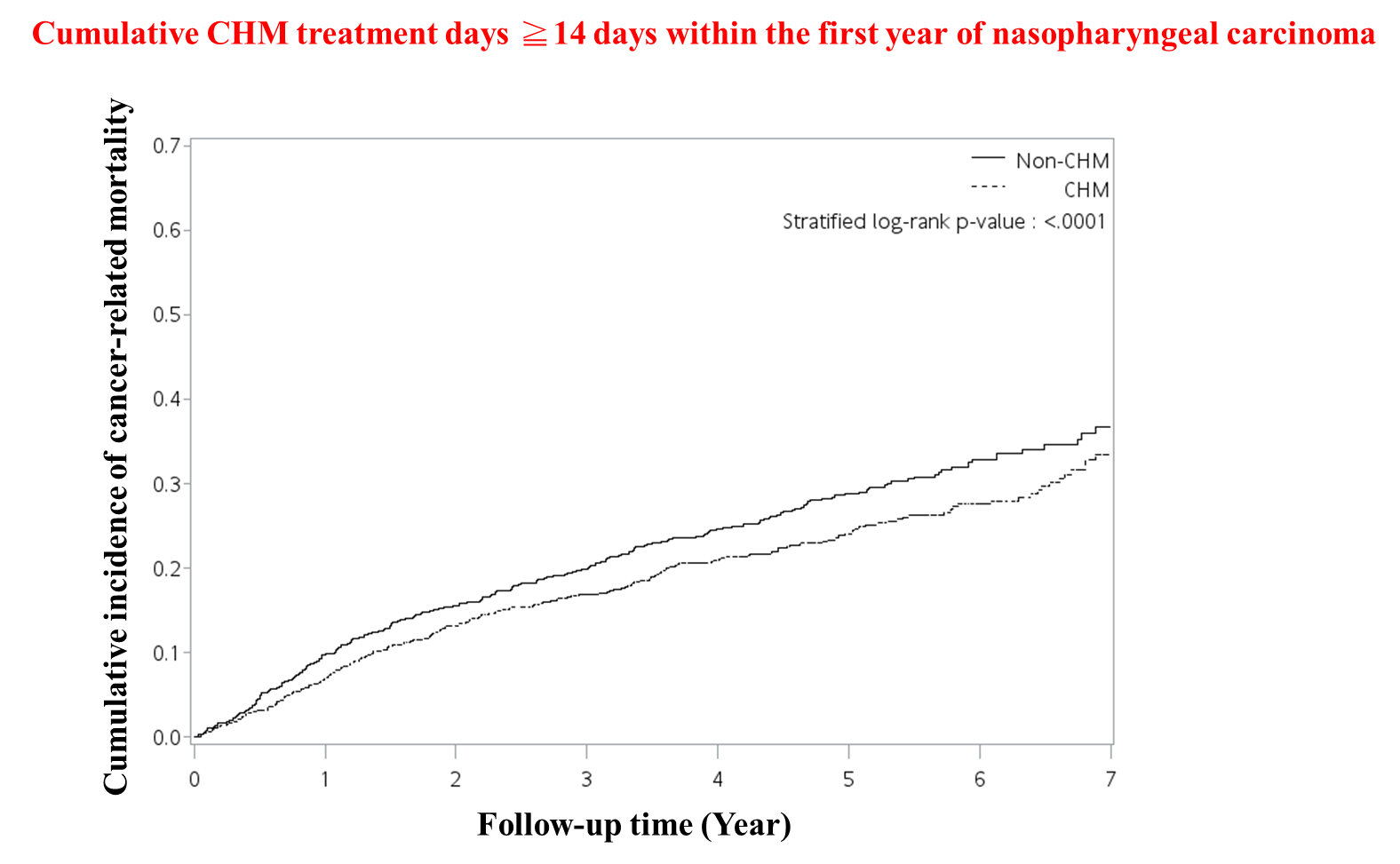


**Fig. S7**


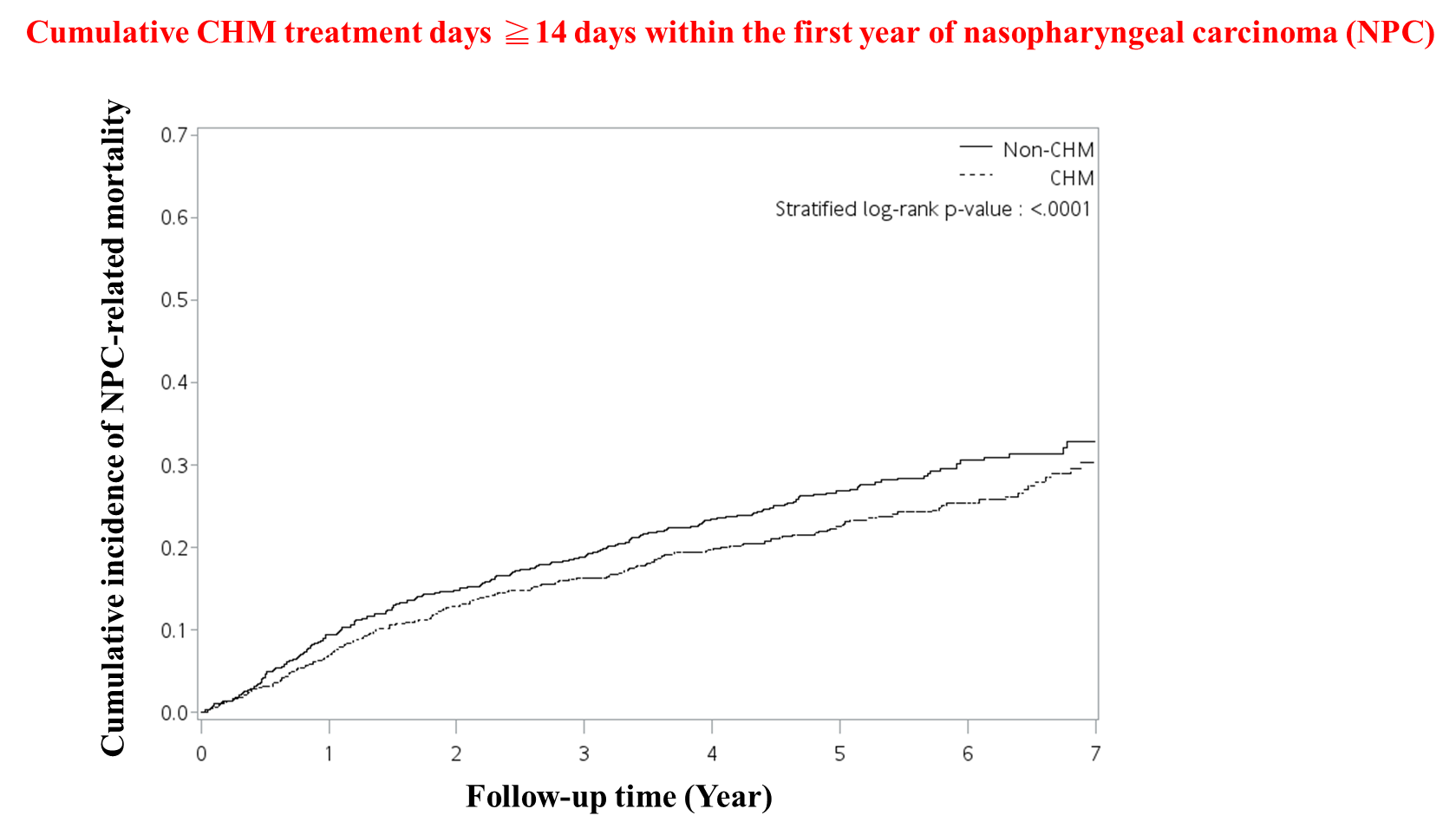


**Fig. S8**


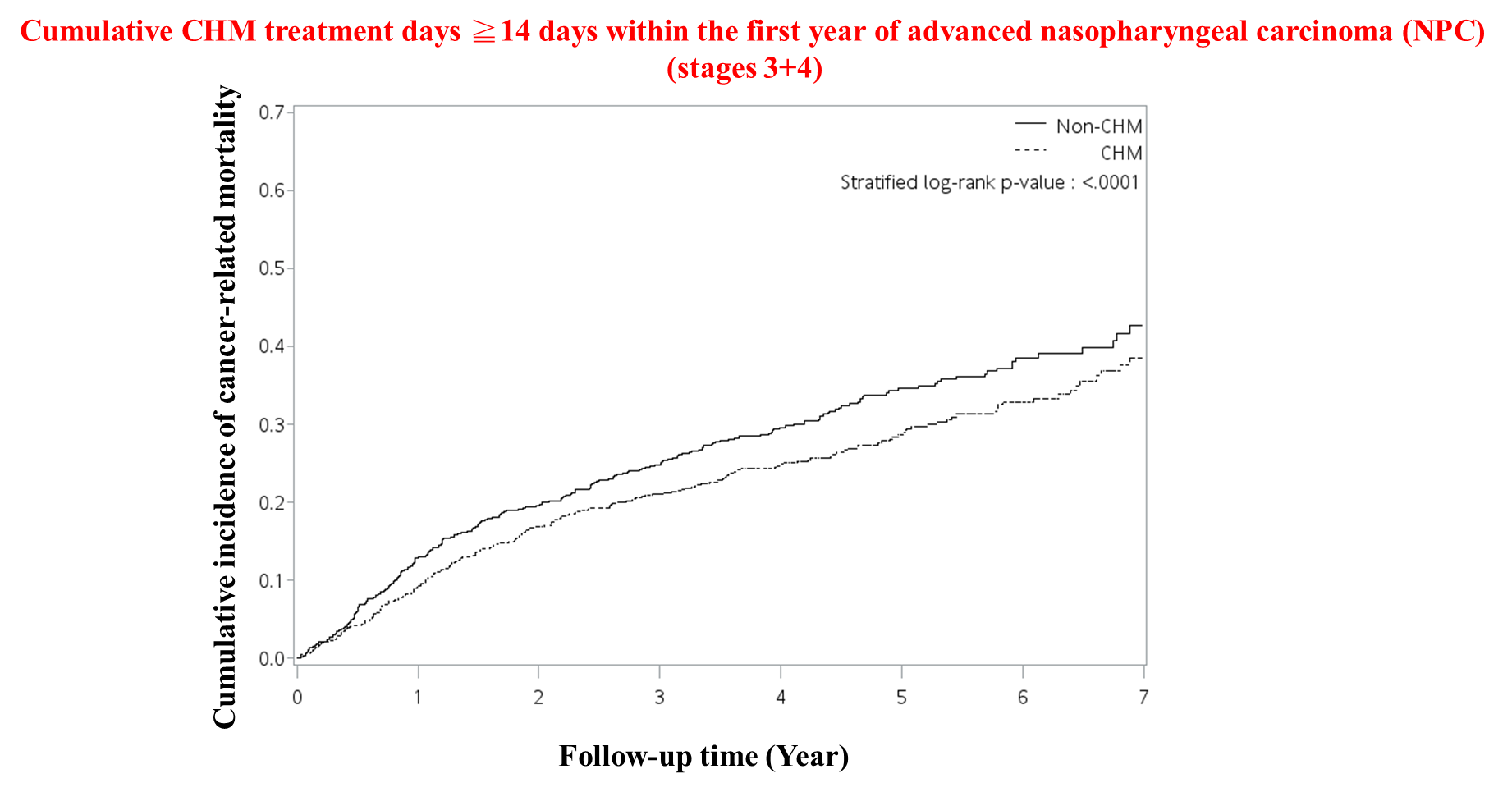


**Fig. S9**


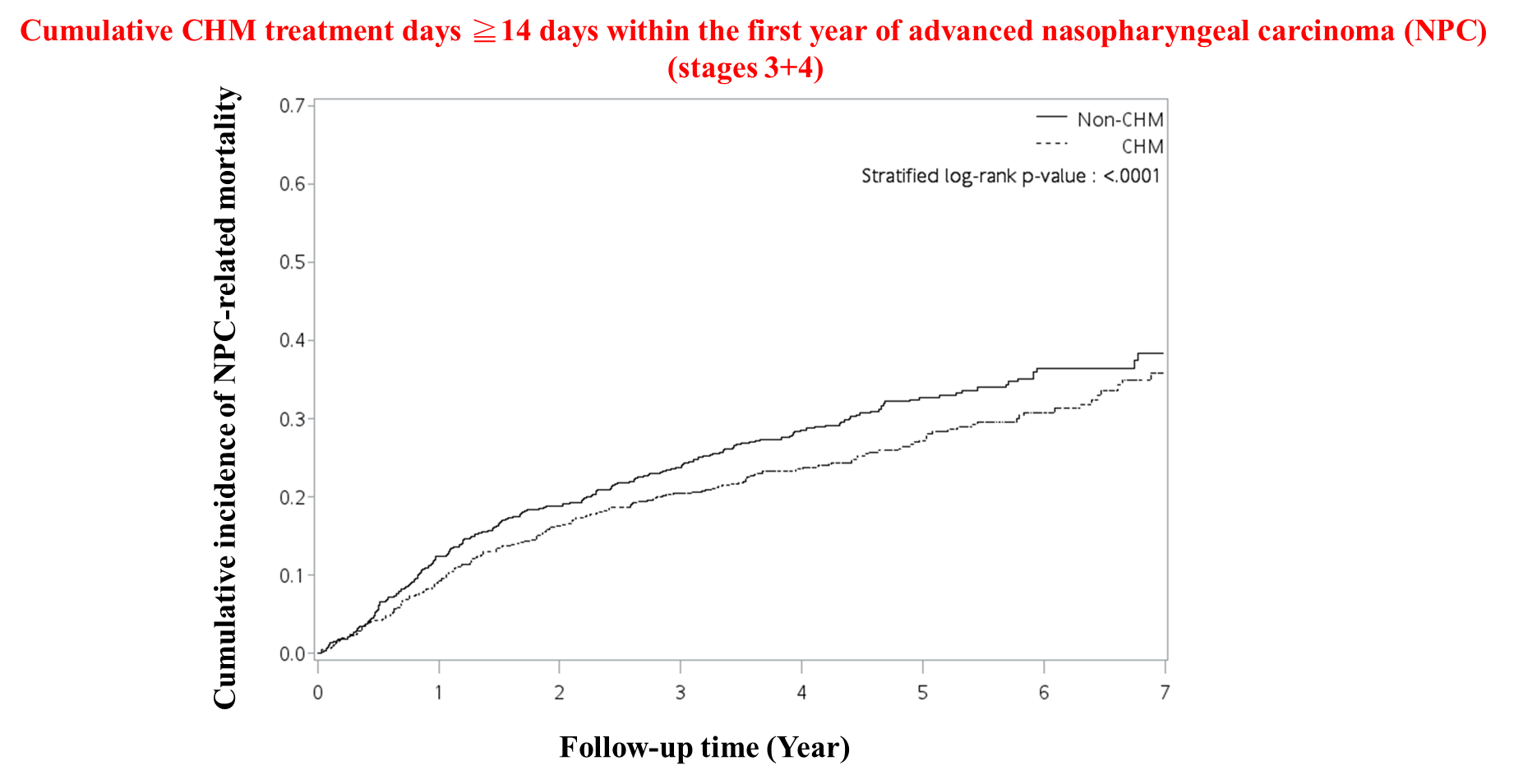


| **Table S1. Composition of herbal formulas and single herbs for patients with nasopharyngeal carcinoma in Taiwan** | | | | | | | | |
| --- | --- | --- | --- | --- | --- | --- | --- | --- |
| **Formulas** | **Chinese name** | **Number of herbs** | **Composition (Pin-yin name (latin name; botanical plant name))** | **Frequency of prescriptions** | **Person-year** | **Percentage of usage person** | **Avg. drug dose per day (g)** | **Average duration for prescription (days)** |
| **Total** |  |  |  | **32842** | **4287.3** | **100.0** | **14.0** | **10.7** |
| **Herbal formula (Pin-yin name)** |  |  |  | **30624** | **4167.3** | **97.4** | **9.5** | **10.5** |
| Gan-Lu-Yin (GLY) | 甘露飲 | 10 | **Sheng-Di-Huang** (*Radix Rehmanniae*; *Thunb.Rehmannia glutinosa (Gaertn.) DC.*), **Shu-Di-Huang** (*Radix Rehmanniae Preparata*; *Thunb.Rehmannia glutinosa (Gaertn.) DC.*), **Shi-Hu** ( *Herba Dendrobii*; *Dendrobium nobile Lindl.*), **Tian-Men-Dong** (*Radix Asparagi*; *Asparagus cochinchinensis (Lour.) Merr.*), **Mai-Men-Dong** ( *Radix Ophiopogonis*; *Ophiopogon japonicus (Thunb.) Ker Gawl.*), **Huang-Qin** (*Radix Scutellariae*; *Scutellaria baicalensis Georgi*), **Yin-Chen** (*Herba Artemisiae Scopariae* ; *Artemisia capillaris Thunb.*), **Zhi-Ke** (*Fructus Aurantii*; *Citrus aurantium L.*), **Pi-Pa-Ye** (*Folium Eriobotryae*; *Eriobotrya japonica (Thunb.) Lindl.*), **Gan-Cao** (*Radix Glycyrrhizae*; *Glycyrrhiza uralensis Fisch.*) | 5185 | 2616.0 | 58.1 | 4.4 | 12.0 |
| Xin-Yi-Qing-Fei-Tang (XYQFT) | 辛夷清肺湯 | 10 | **Xin-Yi** ( *Flos Magnoliae*; *Magnolia biondii Pamp.*), **Pi-Pa-Ye** (*Folium Eriobotryae*; *Eriobotrya japonica (Thunb.) Lindl.*), **Zhi-Zi** (*Fructus Gardeniae*; *Gardenia jasminoides J.Ellis*), **Zhi-Mu** (*Rhizoma Anemarrhenae*; *Anemarrhena asphodeloides Bge.*), **Bai-He** (*Bulbus Lilii*; *Lilium lancifolium Thunb.*), **Huang-Qin** (*Radix Scutellariae*; *Scutellaria baicalensis Georgi*), **Sheng-Ma** (*Rhizoma Cimicifugae*; *Cimicifuga heracleifolia Kom.*), **Mai-Men-Dong** ( *Radix Ophiopogonis*; *Ophiopogon japonicus (Thunb.) Ker Gawl.*), **Shi-Gao** (*Gypsum Fibrosum*), **Gan-Cao** (*Radix Glycyrrhizae*; *Glycyrrhiza uralensis Fisch.*) | 3812 | 1694.5 | 36.1 | 4.3 | 11.5 |
| **Single herbs (Pin-yin name)** |  |  |  | **28934** | **4205.1** | **97.8** | **5.9** | **10.9** |
| Bai-Hua-She-She-Cao (BHSSC) | 白花蛇舌草 | 1 | **Bai-Hua-She-She-Cao** ( *Herba Hedyotis Diffusae; Hedyotis diffusa Willd.*) | 5719 | 1407.3 | 31.8 | 1.0 | 13.8 |
| Xuan-Shen (XS) | 玄參 | 1 | **Xuan-Shen** (*Radix Scrophulariae*; *Scrophularia ningpoensis Hensl.*) | 3323 | 2135.8 | 47.2 | 1.3 | 12.1 |
| Gua-Lou-Gen (GLG) | 栝樓根 | 1 | **Gua-Lou-Gen** (*Radix Trichosanthis*; *Trichosanthes kirilowii Maxim.*) | 3156 | 2023.9 | 44.3 | 1.2 | 14.0 |
| Mai-Men-Dong (MMD) | 麥門冬 | 1 | **Mai-Men-Dong** ( *Radix Ophiopogonis*; *Ophiopogon japonicus (Thunb.) Ker Gawl.*) | 3140 | 1948.6 | 42.6 | 1.2 | 13.2 |
| Sheng-Di-Huang (ShengDH) | 生地黃 | 1 | **Sheng-Di-Huang (***Radix Rehmanniae***;** *Thunb.Rehmannia glutinosa (Gaertn.) DC.***)** | 2232 | 1484.0 | 33.4 | 1.3 | 14.2 |
| Ban-Zhi-Lian (BZL) | 半枝蓮 | 1 | **Ban-Zhi-Lian** (*Herba Scutellariae Barbatae*; *Scutellaria barbata D.Don*) | 2040 | 853.0 | 19.9 | 0.9 | 15.2 |
| *Sorted by frequency of prescriptions. | | | | | | | | |
| Information are obtained from the websites (http://www.americandragon.com/index.htm; http://old.tcmwiki.com/; http://www.shen-nong.com/eng/front/index.html; http://www.ipni.org/; http://www.theplantlist.org/). | | | | | | | | |

| **Table S2. Distribution of the cumulative days of CHM treatment during the study period among patients with advanced nasopharyngeal carcinoma** | | | |
| --- | --- | --- | --- |
| **Cumulative CHM treatment days within the first year after nasopharyngeal carcinoma (NPC)** | **Cumulative CHM treatment days during the study period (started after the index date)** | **CHM users** | |
|  |  | **N** | **%** |
| **≥14 days (N = 992)** |  |  | |
|  | **day < 180** | 540 | 54.4 |
|  | **180 ≤ day< 360** | 163 | 16.4 |
|  | **day ≥ 360** | 289 | 29.1 |
| **≥28 days (N = 876)** |  |  |  |
|  | **day < 180** | 399 | 45.5 |
|  | **180 ≤ day< 360** | 164 | 18.7 |
|  | **day ≥ 360** | 313 | 35.7 |
| **≥56 days (N = 697)** |  |  |  |
|  | **day < 180** | 205 | 29.4 |
|  | **180 ≤ day< 360** | 147 | 21.1 |
|  | **day ≥ 360** | 345 | 49.5 |
| **≥84 days (N = 533)** |  |  |  |
|  | **day < 180** | 94 | 17.6 |
|  | **180 ≤ day< 360** | 128 | 24.0 |
|  | **day ≥ 360** | 311 | 58.3 |
| N, number; CHM, Chinese herbal medicine. | | | |
| The index date of this study was from the day on which the 14, 28, 56, or 84 cumulative days of CHM treatment with the first year were completed. | | | |
| Cumulative CHM treatment days during the study period was started after the index date. | | | |

| **Table S3. Demographic characteristics of patients with nasopharyngeal carcinoma (NPC) (cumulative days of CHM treatment ≧28 days within the first year of NPC)** | | | | | | | |
| --- | --- | --- | --- | --- | --- | --- | --- |
| **Characteristics** | **Total subjects** | | |  | **Matched subjects** | | |
|  | **CHM users** | **Non- users** | ***P*-value** |  | **CHM users** | **Non- users** | ***P*-value** |
|  | **N = 1,149** | **N = 1,128** |  |  | **N = 876** | **N = 876** |  |
|  | **N (%)** | **N (%)** |  |  | **N (%)** | **N (%)** |  |
| **Age (years old)** |  |  | ***0.005*** |  |  |  | 0.836 |
| Age<50 | 624 ( 54.31%) | 536 ( 47.52%) |  |  | 454 ( 51.83%) | 451 ( 51.48%) |  |
| 50≥Age<60 | 344 ( 29.94%) | 380 ( 33.69%) |  |  | 284 ( 32.42%) | 278 ( 31.74%) |  |
| Age≥60 | 181 ( 15.75%) | 212 ( 18.79%) |  |  | 138 ( 15.75%) | 147 ( 16.78%) |  |
| **Gender** |  |  | ***<0.001*** |  |  |  | 0.798 |
| Male | 834 ( 72.58%) | 971 ( 86.08%) |  |  | 731 ( 83.45%) | 727 ( 82.99%) |  |
| Female | 315 ( 27.42%) | 157 ( 13.92%) |  |  | 145 ( 16.55%) | 149 ( 17.01%) |  |
| **CCI score (Mean±SD)** | 0.85± 1.18 | 0.67± 1.11 | ***<0.001*** |  | 0.79± 1.11 | 0.74± 1.18 | 0.451 |
| **T-stage** |  |  | ***<0.001*** |  |  |  | 0.773 |
| T1-T2 | 686 ( 59.7%) | 576 ( 51.06%) |  |  | 496 ( 56.62%) | 490 ( 55.94%) |  |
| T3-T4 | 463 ( 40.3%) | 552 ( 48.94%) |  |  | 380 ( 43.38%) | 386 ( 44.06%) |  |
| **N-stage** |  |  | ***<0.001*** |  |  |  | 0.798 |
| N0 | 133 ( 11.58%) | 123 ( 10.9%) |  |  | 102 ( 11.64%) | 103 ( 11.76%) |  |
| N1-N2 | 860 ( 74.85%) | 766 ( 67.91%) |  |  | 636 ( 72.6%) | 625 ( 71.35%) |  |
| N3-N4 | 156 ( 13.58%) | 239 ( 21.19%) |  |  | 138 ( 15.75%) | 148 ( 16.89%) |  |
| **M-stage** |  |  | ***<0.001*** |  |  |  | 0.476 |
| M0 | 1108 ( 96.43%) | 1048 ( 92.91%) |  |  | 842 ( 96.12%) | 836 ( 95.43%) |  |
| M1 | 41 ( 3.57%) | 80 ( 7.09%) |  |  | 34 ( 3.88%) | 40 ( 4.57%) |  |
| **Cancer stage** |  |  | ***<0.001*** |  |  |  | 0.946 |
| 1 | 39 ( 3.39%) | 31 ( 2.75%) |  |  | 26 ( 2.97%) | 30 ( 3.42%) |  |
| 2 | 314 ( 27.33%) | 231 ( 20.48%) |  |  | 210 ( 23.97%) | 214 ( 24.43%) |  |
| **3** | 448 ( 38.99%) | 381 ( 33.78%) |  |  | 332 ( 37.9%) | 328 ( 37.44%) |  |
| 4 | 348 ( 30.29%) | 485 ( 43%) |  |  | 308 ( 35.16%) | 304 ( 34.7%) |  |
| **Surgery** |  |  | 0.059 |  |  |  | 0.426 |
| No | 1105 ( 96.17%) | 1066 ( 94.5%) |  |  | 839 ( 95.78%) | 832 ( 94.98%) |  |
| Yes | 44 ( 3.83%) | 62 ( 5.5%) |  |  | 37 ( 4.22%) | 44 ( 5.02%) |  |
| N, number; CHM, Chinese herbal medicine; CCI, Charlson comorbidity index; T-stage, tumor stage; N-stage, lymph nodes stage; M-stage, metastasis stage. | | | | | | | |
| Age, gender, TNM stage, cancer stage, and surgery were expressed as categorical variable (number (%)). | | | | | | | |
| Nasopharyngeal carcinoma (ICD-9-CM-code: 147). | | | | | | | |
| *P*-values were obtained by chi-square test. Significant *p-*values (*p* < 0.05) were highlighted in bold italic. | | | | | | | |
| Propensity score matching was performed for age, gender, CCI score, and cancer stage (1:1 ratio). | | | | | | | |
| The Charlson comorbidities include congestive heart failure (ICD-9-CM: 398.91, 402.01, 402.11, 402.91, 404.01, 404.03, 404.11, 404.13, 404.91, 404.93, 425.4 - 425.9, 428.x), peripheral vascular disease (ICD-9-CM: 093.0, 437.3, 440.x, 441.x, 443.1 - 443.9, 447.1, 557.1, 557.9, V43.4), cerebrovascular disease (ICD-9-CM: 362.34, 430.x - 438.x), chronic pulmonary disease (ICD-9-CM: 416.8, 416.9, 490.x - 505.x, 506.4, 508.1, 508.8), rheumatic disease (ICD-9-CM: 446.5, 710.0 - 710.4, 714.0 - 714.2, 714.8, 725.x), peptic ulcer disease (ICD-9-CM: 531.x - 534.x), mild liver disease (ICD-9-CM: 070.22, 070.23, 070.32, 070.33, 070.44, 070.54, 070.6, 070.9, 570.x, 571.x, 573.3, 573.4, 573.8, 573.9, V42.7), diabetes without chronic complication (ICD-9-CM: 250.0 - 250.3, 250.8, 250.9), diabetes with chronic complication (ICD-9-CM: 250.4 - 250.7), hemiplegia or paraplegia (ICD-9-CM: 334.1, 342.x, 343.x, 344.0 - 344.6, 344.9), renal disease (ICD-9-CM: 403.01, 403.11, 403.91, 404.02, 404.03, 404.12, 404.13, 404.92, 404.93, 582.x, 583.0 - 583.7, 585.x, 586.x, 588.0, V42.0, V45.1, V56.x), and any malignancy, including lymphoma and leukemia, except malignant neoplasm of skin (ICD-9-CM: 140.x - 172.x, 174.x - 195.8, 200.x - 208.x, 238.6). These comorbidities were recorded before the diagnosis of nasopharyngeal carcinoma. | | | | | | | |

| **Table S4. Cox proportional hazard models for overall mortality in patients with nasopharyngeal carcinoma (NPC) in Taiwan (cumulative days of CHM treatment ≧28 days within the first year of NPC)** | | | | | | | |
| --- | --- | --- | --- | --- | --- | --- | --- |
|  | **Crude** | | |  | **Adjusted** | | |
|  | **HR** | **(95% CI)** | ***P*-value** |  | **aHR** | **(95% CI)** | ***P*-value** |
| **Age (years old)** |  |  |  |  |  |  |  |
| Age<50 | Ref. | ND | ND |  | Ref. | ND | ND |
| 50≥Age<60 | 1.25 | (1.02-1.54) | ***0.0357*** |  | 1.255 | (1.01-1.55) | ***0.0381*** |
| Age≥60 | 2.514 | (2.01-3.14) | ***<.0001*** |  | 2.364 | (1.86-3.01) | ***<.0001*** |
| **Gender** |  |  |  |  |  |  |  |
| Male | Ref. | ND | ND |  | Ref. | ND | ND |
| Female | 0.882 | (0.69-1.13) | 0.3192 |  | 0.806 | (0.63-1.04) | 0.097 |
| **CHM use** |  |  |  |  |  |  |  |
| No | Ref. | ND | ND |  | Ref. | ND | ND |
| Yes | 0.823 | (0.7-0.97) | ***0.0232*** |  | 0.783 | (0.66-0.94) | ***0.007*** |
| **CCI score (Mean±SD), per score** | 1.211 | (1.13-1.3) | ***<.0001*** |  | 1.088 | (1.01-1.17) | ***0.0227*** |
| **Cancer stage** |  |  |  |  |  |  |  |
| 1 | Ref. | ND | ND |  | Ref. | ND | ND |
| 2 | 1.214 | (0.56-2.64) | 0.6251 |  | 1.237 | (0.58-2.64) | 0.5831 |
| 3 | 2.005 | (0.94-4.27) | 0.071 |  | 2.089 | (1-4.35) | ***0.049*** |
| 4 | 4.711 | (2.22-10.01) | ***<.0001*** |  | 4.91 | (2.36-10.23) | ***<.0001*** |
| **Surgery** |  |  |  |  |  |  |  |
| No | Ref. | ND | ND |  | Ref. | ND | ND |
| Yes | 0.993 | (0.68-1.46) | 0.9707 |  | 0.879 | (0.58-1.33) | 0.5402 |
| CHM, Chinese herbal medicine; HR, hazard ratio; 95% CI, 95% confidence interval; ND, not determined; CCI, Charlson comorbidity index. | | | | | | | |
| Nasopharyngeal carcinoma (ICD-9-CM-code: 147). | | | | | | | |
| CCI score (Mean±SD) was expressed as a continuous variable. The risk of overall mortality increased with CCI score (HR 1.088/score) in our study. | | | | | | | |
| Models adjusted for age, gender, CHM use, CCI score, cancer stage, and surgery. | | | | | | | |
| *P*-value (*p* < 0.05) was shown in bold italic font. | | | | | | | |

| **Table S5. Demographic characteristics of patients with nasopharyngeal carcinoma (NPC) (cumulative days of CHM treatment ≧56 days within the first year of NPC)** | | | | | | | |
| --- | --- | --- | --- | --- | --- | --- | --- |
| **Characteristics** | **Total subjects** | | |  | **Matched subjects** | | |
|  | **CHM users** | **Non- users** | ***P*-value** |  | **CHM users** | **Non- users** | ***P*-value** |
|  | **N = 791** | **N = 1,128** |  |  | **N = 697** | **N = 697** |  |
|  | **N (%)** | **N (%)** |  |  | **N (%)** | **N (%)** |  |
| **Age (years old)** |  |  | ***0.002*** |  |  |  | 0.552 |
| Age<50 | 439 ( 55.5%) | 536 ( 47.52%) |  |  | 380 ( 54.52%) | 370 ( 53.08%) |  |
| 50≥Age<60 | 231 ( 29.2%) | 380 ( 33.69%) |  |  | 205 ( 29.41%) | 223 ( 31.99%) |  |
| Age≥60 | 121 ( 15.3%) | 212 ( 18.79%) |  |  | 112 ( 16.07%) | 104 ( 14.92%) |  |
| **Gender** |  |  | ***<0.001*** |  |  |  | 0.689 |
| Male | 579 ( 73.2%) | 971 ( 86.08%) |  |  | 553 ( 79.34%) | 559 ( 80.2%) |  |
| Female | 212 ( 26.8%) | 157 ( 13.92%) |  |  | 144 ( 20.66%) | 138 ( 19.8%) |  |
| **CCI score (Mean±SD)** | 0.83± 1.11 | 0.67± 1.11 | ***0.002*** |  | 0.8± 1.05 | 0.78± 1.21 | 0.850 |
| **T-stage** |  |  | ***<0.001*** |  |  |  | 0.745 |
| T1-T2 | 467 ( 59.04%) | 576 ( 51.06%) |  |  | 403 ( 57.82%) | 409 ( 58.68%) |  |
| T3-T4 | 324 ( 40.96%) | 552 ( 48.94%) |  |  | 294 ( 42.18%) | 288 ( 41.32%) |  |
| **N-stage** |  |  | ***<0.001*** |  |  |  | 0.470 |
| N0 | 87 ( 11%) | 123 ( 10.9%) |  |  | 79 ( 11.33%) | 86 ( 12.34%) |  |
| N1-N2 | 601 ( 75.98%) | 766 ( 67.91%) |  |  | 522 ( 74.89%) | 502 ( 72.02%) |  |
| N3-N4 | 103 ( 13.02%) | 239 ( 21.19%) |  |  | 96 ( 13.77%) | 109 ( 15.64%) |  |
| **M-stage** |  |  | ***<0.001*** |  |  |  | 0.084 |
| M0 | 765 ( 96.71%) | 1048 ( 92.91%) |  |  | 674 ( 96.7%) | 661 ( 94.84%) |  |
| M1 | 26 ( 3.29%) | 80 ( 7.09%) |  |  | 23 ( 3.3%) | 36 ( 5.16%) |  |
| **Cancer stage** |  |  | ***<0.001*** |  |  |  | 0.814 |
| 1 | 24 ( 3.03%) | 31 ( 2.75%) |  |  | 22 ( 3.16%) | 26 ( 3.73%) |  |
| 2 | 220 ( 27.81%) | 231 ( 20.48%) |  |  | 180 ( 25.82%) | 191 ( 27.4%) |  |
| 3 | 310 ( 39.19%) | 381 ( 33.78%) |  |  | 277 ( 39.74%) | 265 ( 38.02%) |  |
| 4 | 237 ( 29.96%) | 485 ( 43%) |  |  | 218 ( 31.28%) | 215 ( 30.85%) |  |
| **Surgery** |  |  | 0.147 |  |  |  | 0.443 |
| No | 759 ( 95.95%) | 1066 ( 94.5%) |  |  | 668 ( 95.84%) | 662 ( 94.98%) |  |
| Yes | 32 ( 4.05%) | 62 ( 5.5%) |  |  | 29 ( 4.16%) | 35 ( 5.02%) |  |
| N, number; CHM, Chinese herbal medicine; CCI, Charlson comorbidity index; T-stage, tumor stage; N-stage, lymph nodes stage; M-stage, metastasis stage. | | | | | | | |
| Age, gender, TNM stage, cancer stage, and surgery were expressed as categorical variable (number (%)). | | | | | | | |
| Nasopharyngeal carcinoma (ICD-9-CM-code: 147). | | | | | | | |
| *P*-values were obtained by chi-square test. Significant *p-*values (*p* < 0.05) were highlighted in bold italic. | | | | | | | |
| Propensity score matching was performed for age, gender, CCI score, and cancer stage (1:1 ratio). | | | | | | | |
| The Charlson comorbidities include congestive heart failure (ICD-9-CM: 398.91, 402.01, 402.11, 402.91, 404.01, 404.03, 404.11, 404.13, 404.91, 404.93, 425.4 - 425.9, 428.x), peripheral vascular disease (ICD-9-CM: 093.0, 437.3, 440.x, 441.x, 443.1 - 443.9, 447.1, 557.1, 557.9, V43.4), cerebrovascular disease (ICD-9-CM: 362.34, 430.x - 438.x), chronic pulmonary disease (ICD-9-CM: 416.8, 416.9, 490.x - 505.x, 506.4, 508.1, 508.8), rheumatic disease (ICD-9-CM: 446.5, 710.0 - 710.4, 714.0 - 714.2, 714.8, 725.x), peptic ulcer disease (ICD-9-CM: 531.x - 534.x), mild liver disease (ICD-9-CM: 070.22, 070.23, 070.32, 070.33, 070.44, 070.54, 070.6, 070.9, 570.x, 571.x, 573.3, 573.4, 573.8, 573.9, V42.7), diabetes without chronic complication (ICD-9-CM: 250.0 - 250.3, 250.8, 250.9), diabetes with chronic complication (ICD-9-CM: 250.4 - 250.7), hemiplegia or paraplegia (ICD-9-CM: 334.1, 342.x, 343.x, 344.0 - 344.6, 344.9), renal disease (ICD-9-CM: 403.01, 403.11, 403.91, 404.02, 404.03, 404.12, 404.13, 404.92, 404.93, 582.x, 583.0 - 583.7, 585.x, 586.x, 588.0, V42.0, V45.1, V56.x), and any malignancy, including lymphoma and leukemia, except malignant neoplasm of skin (ICD-9-CM: 140.x - 172.x, 174.x - 195.8, 200.x - 208.x, 238.6). These comorbidities were recorded before the diagnosis of nasopharyngeal carcinoma. | | | | | | | |

| **Table S6. Cox proportional hazard models for overall mortality in patients with nasopharyngeal carcinoma (NPC) in Taiwan (cumulative days of CHM treatment ≧56 days within the first year of NPC)** | | | | | | | |
| --- | --- | --- | --- | --- | --- | --- | --- |
|  | **Crude** | | |  | **Adjusted** | | |
|  | **HR** | **(95% CI)** | ***P*-value** |  | **aHR** | **(95% CI)** | ***P*-value** |
| **Age (years old)** |  |  |  |  |  |  |  |
| Age<50 | Ref. | ND | ND |  | Ref. | ND | ND |
| 50≥Age<60 | 1.348 | (1.06-1.71) | ***0.0144*** |  | 1.308 | (1.02-1.68) | ***0.0363*** |
| Age≥60 | 2.558 | (1.99-3.29) | ***<.0001*** |  | 2.363 | (1.79-3.13) | ***<.0001*** |
| **Gender** |  |  |  |  |  |  |  |
| Male | Ref. | ND | ND |  | Ref. | ND | ND |
| Female | 0.778 | (0.58-1.04) | 0.0946 |  | 0.677 | (0.51-0.91) | ***0.0092*** |
| **CHM use** |  |  |  |  |  |  |  |
| No | Ref. | ND | ND |  | Ref. | ND | ND |
| Yes | 0.827 | (0.68-1.01) | 0.0583 |  | 0.771 | (0.62-0.95) | ***0.0157*** |
| **CCI score (Mean±SD), per score** | 1.252 | (1.16-1.36) | ***<.0001*** |  | 1.127 | (1.03-1.23) | ***0.0066*** |
| **Cancer stage** |  |  |  |  |  |  |  |
| 1 | Ref. | ND | ND |  | Ref. | ND | ND |
| 2 | 1.813 | (0.65-5.03) | 0.2525 |  | 1.818 | (0.67-4.96) | 0.2427 |
| 3 | 2.886 | (1.07-7.81) | ***0.0369*** |  | 3.039 | (1.14-8.1) | ***0.0263*** |
| 4 | 6.731 | (2.5-18.15) | ***0.0002*** |  | 7.198 | (2.71-19.15) | ***<.0001*** |
| **Surgery** |  |  |  |  |  |  |  |
| No | Ref. | ND | ND |  | Ref. | ND | ND |
| Yes | 0.87 | (0.54-1.4) | 0.5647 |  | 0.742 | (0.45-1.22) | 0.2417 |
| CHM, Chinese herbal medicine; HR, hazard ratio; 95% CI, 95% confidence interval; ND, not determined; CCI, Charlson comorbidity index. | | | | | | | |
| Nasopharyngeal carcinoma (ICD-9-CM-code: 147). | | | | | | | |
| CCI score (Mean±SD) was expressed as a continuous variable. The risk of overall mortality increased with CCI score (HR 1.127/score) in our study. | | | | | | | |
| Models adjusted for age, gender, CHM use, CCI score, cancer stage, and surgery. | | | | | | | |
| *P*-value (*p* < 0.05) was shown in bold italic font. | | | | | | | |

| **Table S7. Demographic characteristics of patients with nasopharyngeal carcinoma (NPC) (cumulative days of CHM treatment ≧84 days within the first year of NPC)** | | | | | | | |
| --- | --- | --- | --- | --- | --- | --- | --- |
| **Characteristics** | **Total subjects** | | |  | **Matched subjects** | | |
|  | **CHM users** | **Non- users** | ***P*-value** |  | **CHM users** | **Non- users** | ***P*-value** |
|  | **N = 566** | **N = 1,128** |  |  | **N = 533** | **N = 533** |  |
|  | **N (%)** | **N (%)** |  |  | **N (%)** | **N (%)** |  |
| **Age (years old)** |  |  | ***0.006*** |  |  |  | 0.441 |
| Age<50 | 315 ( 55.65%) | 536 ( 47.52%) |  |  | 295 ( 55.35%) | 276 ( 51.78%) |  |
| 50≥Age<60 | 164 ( 28.98%) | 380 ( 33.69%) |  |  | 153 ( 28.71%) | 171 ( 32.08%) |  |
| Age≥60 | 87 ( 15.37%) | 212 ( 18.79%) |  |  | 85 ( 15.95%) | 86 ( 16.14%) |  |
| **Gender** |  |  | ***<0.001*** |  |  |  | 0.560 |
| Male | 422 ( 74.56%) | 971 ( 86.08%) |  |  | 407 ( 76.36%) | 415 ( 77.86%) |  |
| Female | 144 ( 25.44%) | 157 ( 13.92%) |  |  | 126 ( 23.64%) | 118 ( 22.14%) |  |
| **CCI score (Mean±SD)** | 0.82± 1.11 | 0.67± 1.11 | ***0.013*** |  | 0.81± 1.11 | 0.79± 1.21 | 0.752 |
| **T-stage** |  |  | ***0.013*** |  |  |  | 0.493 |
| T1-T2 | 325 ( 57.42%) | 576 ( 51.06%) |  |  | 309 ( 57.97%) | 320 ( 60.04%) |  |
| T3-T4 | 241 ( 42.58%) | 552 ( 48.94%) |  |  | 224 ( 42.03%) | 213 ( 39.96%) |  |
| **N-stage** |  |  | ***<0.001*** |  |  |  | 0.097 |
| N0 | 62 ( 10.95%) | 123 ( 10.9%) |  |  | 57 ( 10.69%) | 79 ( 14.82%) |  |
| N1-N2 | 431 ( 76.15%) | 766 ( 67.91%) |  |  | 407 ( 76.36%) | 380 ( 71.29%) |  |
| N3-N4 | 73 ( 12.9%) | 239 ( 21.19%) |  |  | 69 ( 12.95%) | 74 ( 13.88%) |  |
| **M-stage** |  |  | ***0.003*** |  |  |  | 0.519 |
| M0 | 546 ( 96.47%) | 1048 ( 92.91%) |  |  | 515 ( 96.62%) | 511 ( 95.87%) |  |
| M1 | 20 ( 3.53%) | 80 ( 7.09%) |  |  | 18 ( 3.38%) | 22 ( 4.13%) |  |
| **Cancer stage** |  |  | ***<0.001*** |  |  |  | 0.653 |
| 1 | 17 ( 3%) | 31 ( 2.75%) |  |  | 17 ( 3.19%) | 23 ( 4.32%) |  |
| 2 | 155 ( 27.39%) | 231 ( 20.48%) |  |  | 147 ( 27.58%) | 152 ( 28.52%) |  |
| 3 | 222 ( 39.22%) | 381 ( 33.78%) |  |  | 205 ( 38.46%) | 208 ( 39.02%) |  |
| 4 | 172 ( 30.39%) | 485 ( 43%) |  |  | 164 ( 30.77%) | 150 ( 28.14%) |  |
| **Surgery** |  |  | 0.150 |  |  |  | 0.465 |
| No | 544 ( 96.11%) | 1066 ( 94.5%) |  |  | 511 ( 95.87%) | 506 ( 94.93%) |  |
| Yes | 22 ( 3.89%) | 62 ( 5.5%) |  |  | 22 ( 4.13%) | 27 ( 5.07%) |  |
| N, number; CHM, Chinese herbal medicine; CCI, Charlson comorbidity index; T-stage, tumor stage; N-stage, lymph nodes stage; M-stage, metastasis stage. | | | | | | | |
| Age, gender, TNM stage, cancer stage, and surgery were expressed as categorical variable (number (%)). | | | | | | | |
| Nasopharyngeal carcinoma (ICD-9-CM-code: 147). | | | | | | | |
| *P*-values were obtained by chi-square test. Significant *p-*values (*p* < 0.05) were highlighted in bold italic. | | | | | | | |
| Propensity score matching was performed for age, gender, CCI score, and cancer stage (1:1 ratio). | | | | | | | |
| The Charlson comorbidities include congestive heart failure (ICD-9-CM: 398.91, 402.01, 402.11, 402.91, 404.01, 404.03, 404.11, 404.13, 404.91, 404.93, 425.4 - 425.9, 428.x), peripheral vascular disease (ICD-9-CM: 093.0, 437.3, 440.x, 441.x, 443.1 - 443.9, 447.1, 557.1, 557.9, V43.4), cerebrovascular disease (ICD-9-CM: 362.34, 430.x - 438.x), chronic pulmonary disease (ICD-9-CM: 416.8, 416.9, 490.x - 505.x, 506.4, 508.1, 508.8), rheumatic disease (ICD-9-CM: 446.5, 710.0 - 710.4, 714.0 - 714.2, 714.8, 725.x), peptic ulcer disease (ICD-9-CM: 531.x - 534.x), mild liver disease (ICD-9-CM: 070.22, 070.23, 070.32, 070.33, 070.44, 070.54, 070.6, 070.9, 570.x, 571.x, 573.3, 573.4, 573.8, 573.9, V42.7), diabetes without chronic complication (ICD-9-CM: 250.0 - 250.3, 250.8, 250.9), diabetes with chronic complication (ICD-9-CM: 250.4 - 250.7), hemiplegia or paraplegia (ICD-9-CM: 334.1, 342.x, 343.x, 344.0 - 344.6, 344.9), renal disease (ICD-9-CM: 403.01, 403.11, 403.91, 404.02, 404.03, 404.12, 404.13, 404.92, 404.93, 582.x, 583.0 - 583.7, 585.x, 586.x, 588.0, V42.0, V45.1, V56.x), and any malignancy, including lymphoma and leukemia, except malignant neoplasm of skin (ICD-9-CM: 140.x - 172.x, 174.x - 195.8, 200.x - 208.x, 238.6). These comorbidities were recorded before the diagnosis of nasopharyngeal carcinoma. | | | | | | | |

| **Table S8. Cox proportional hazard models for overall mortality in patients with nasopharyngeal carcinoma (NPC) in Taiwan (cumulative days of CHM treatment ≧84 days within the first year of NPC)** | | | | | | | |
| --- | --- | --- | --- | --- | --- | --- | --- |
|  | **Crude** | | |  | **Adjusted** | | |
|  | **HR** | **(95% CI)** | ***P*-value** |  | **aHR** | **(95% CI)** | ***P*-value** |
| **Age (years old)** |  |  |  |  |  |  |  |
| Age<50 | Ref. | ND | ND |  | Ref. | ND | ND |
| 50≥Age<60 | 1.522 | (1.15-2.02) | ***0.0038*** |  | 1.442 | (1.07-1.94) | ***0.0152*** |
| Age≥60 | 2.507 | (1.85-3.4) | ***<.0001*** |  | 2.353 | (1.68-3.31) | ***<.0001*** |
| **Gender** |  |  |  |  |  |  |  |
| Male | Ref. | ND | ND |  | Ref. | ND | ND |
| Female | 0.851 | (0.63-1.14) | 0.2845 |  | 0.869 | (0.65-1.17) | 0.351 |
| **CHM use** |  |  |  |  |  |  |  |
| No | Ref. | ND | ND |  | Ref. | ND | ND |
| Yes | 0.797 | (0.63-1.01) | 0.0551 |  | 0.772 | (0.61-0.98) | ***0.0353*** |
| **CCI score (Mean±SD), per score** | 1.22 | (1.11-1.34) | ***<.0001*** |  | 1.125 | (1.01-1.25) | ***0.0259*** |
| **Cancer stage** |  |  |  |  |  |  |  |
| 1 | Ref. | ND | ND |  | Ref. | ND | ND |
| 2 | 1.227 | (0.5-3.04) | 0.6589 |  | 1.204 | (0.5-2.88) | 0.6766 |
| 3 | 1.85 | (0.76-4.49) | 0.1735 |  | 1.894 | (0.81-4.41) | 0.1383 |
| 4 | 4.32 | (1.79-10.44) | ***0.0011*** |  | 4.529 | (1.95-10.5) | ***0.0004*** |
| **Surgery** |  |  |  |  |  |  |  |
| No | Ref. | ND | ND |  | Ref. | ND | ND |
| Yes | 1.192 | (0.7-2.04) | 0.5186 |  | 1.075 | (0.63-1.84) | 0.7928 |
| CHM, Chinese herbal medicine; HR, hazard ratio; 95% CI, 95% confidence interval; ND, not determined; CCI, Charlson comorbidity index. | | | | | | | |
| Nasopharyngeal carcinoma (ICD-9-CM-code: 147). | | | | | | | |
| CCI score (Mean±SD) was expressed as a continuous variable. The risk of overall mortality increased with CCI score (HR 1.125/score) in our study. | | | | | | | |
| Models adjusted for age, gender, CHM use, CCI score, cancer stage, and surgery. | | | | | | | |
| *P*-value (*p* < 0.05) was shown in bold italic font. | | | | | | | |

| **Table S9. Cox proportional hazard models for cancer-related mortality in patients with nasopharyngeal carcinoma (NPC) in Taiwan (cumulative days of CHM treatment ≧14 days within the first year of NPC)** | | | | | | | |
| --- | --- | --- | --- | --- | --- | --- | --- |
|  | **Crude** | | |  | **Adjusted** | | |
|  | **HR** | **(95% CI)** | ***P*-value** |  | **aHR** | **(95% CI)** | ***P*-value** |
| **Age (years old)** |  |  |  |  |  |  |  |
| Age<50 | Ref. | ND | ND |  | Ref. | ND | ND |
| 50≥Age<60 | 1.18 | (0.96-1.44) | 0.1178 |  | 1.15 | (0.94-1.42) | 0.18 |
| Age≥60 | 2.11 | (1.70-2.62) | ***<0.001*** |  | 2.05 | (1.63-2.58) | ***<0.001*** |
| **Gender** |  |  |  |  |  |  |  |
| Male | Ref. | ND | ND |  | Ref. | ND | ND |
| Female | 0.69 | (0.52-0.91) | ***0.0096*** |  | 0.68 | (0.52-0.91) | ***0.0081*** |
| **CHM use** |  |  |  |  |  |  |  |
| No | Ref. | ND | ND |  | Ref. | ND | ND |
| Yes | 0.84 | (0.71-0.98) | ***0.0291*** |  | 0.83 | (0.70-0.97) | ***0.0214*** |
| **CCI score (Mean±SD), per score** | 1.11 | (1.03-1.20) | ***0.0063*** |  | 1.03 | (0.95-1.12) | 0.4735 |
| **Surgery** |  |  |  |  |  |  |  |
| No | Ref. | ND | ND |  | Ref. | ND | ND |
| Yes | 1.25 | (0.88-1.78) | 0.2139 |  | 1.20 | (0.84-1.72) | 0.3082 |
| CHM, Chinese herbal medicine; HR, hazard ratio; 95% CI, 95% confidence interval; ND, not determined; CCI, Charlson comorbidity index. | | | | | | | |
| Nasopharyngeal carcinoma (NPC) (ICD-9-CM-code: 147). | | | | | | | |
| CCI score (Mean±SD) was expressed as a continuous variable. | | | | | | | |
| Models adjusted for age, gender, CHM use, CCI score, and surgery. | | | | | | | |
| *P*-value (*p* < 0.05) was shown in bold italic font. | | | | | | | |

| **Table S10. Cox proportional hazard models for NPC-related mortality in patients with nasopharyngeal carcinoma (NPC) in Taiwan (cumulative days of CHM treatment ≧14 days within the first year of NPC)** | | | | | | | |
| --- | --- | --- | --- | --- | --- | --- | --- |
|  | **Crude** | | |  | **Adjusted** | | |
|  | **HR** | **(95% CI)** | ***P*-value** |  | **aHR** | **(95% CI)** | ***P*-value** |
| **Age (years old)** |  |  |  |  |  |  |  |
| Age<50 | Ref. | ND | ND |  | Ref. | ND | ND |
| 50≥Age<60 | 1.11 | (0.89-1.37) | 0.3547 |  | 1.08 | (0.87-1.34) | 0.4682 |
| Age≥60 | 2.01 | (1.61-2.52) | ***<0.001*** |  | 1.95 | (1.54-2.48) | ***<0.001*** |
| **Gender** |  |  |  |  |  |  |  |
| Male | Ref. | ND | ND |  | Ref. | ND | ND |
| Female | 0.72 | (0.54-0.95) | ***0.0227*** |  | 0.71 | (0.53-0.95) | ***0.0194*** |
| **CHM use** |  |  |  |  |  |  |  |
| No | Ref. | ND | ND |  | Ref. | ND | ND |
| Yes | 0.84 | (0.71-1.00) | ***0.0461*** |  | 0.83 | (0.70-0.99) | ***0.0352*** |
| **CCI score (Mean±SD), per score** | 1.11 | (1.02-1.20) | ***0.0108*** |  | 1.03 | (0.95-1.12) | 0.4713 |
| **Surgery** |  |  |  |  |  |  |  |
| No | Ref. | ND | ND |  | Ref. | ND | ND |
| Yes | 1.21 | (0.84-1.75) | 0.2969 |  | 1.17 | (0.81-1.69) | 0.3998 |
| CHM, Chinese herbal medicine; HR, hazard ratio; 95% CI, 95% confidence interval; ND, not determined; CCI, Charlson comorbidity index. | | | | | | | |
| Nasopharyngeal carcinoma (NPC) (ICD-9-CM-code: 147). | | | | | | | |
| CCI score (Mean±SD) was expressed as a continuous variable. | | | | | | | |
| Models adjusted for age, gender, CHM use, CCI score, and surgery. | | | | | | | |
| *P*-value (*p* < 0.05) was shown in bold italic font. | | | | | | | |

| **Table S11.** Cox proportional hazard models for cancer-related mortality in patients with advanced nasopharyngeal carcinoma (NPC) (stages 3+4) in Taiwan (cumulative days of CHM treatment ≧14 days within the first year of advanced NPC) | | | | | | | |
| --- | --- | --- | --- | --- | --- | --- | --- |
|  | **Crude** | | |  | **Adjusted** | | |
|  | **HR** | **(95% CI)** | ***P*-value** |  | **aHR** | **(95% CI)** | ***P*-value** |
| **Age (years old)** |  |  |  |  |  |  |  |
| Age<50 | Ref. | ND | ND |  | Ref. | ND | ND |
| 50≥Age<60 | 1.24 | (1.00-1.55) | ***0.048*** |  | 1.24 | (0.99-1.54) | 0.0561 |
| Age≥60 | 2.15 | (1.71-2.71) | ***<0.001*** |  | 2.18 | (1.71-2.80) | ***<0.001*** |
| **Gender** |  |  |  |  |  |  |  |
| Male | Ref. | ND | ND |  | Ref. | ND | ND |
| Female | 0.70 | (0.52-0.95) | ***0.0215*** |  | 0.71 | (0.53-0.96) | ***0.0268*** |
| **CHM use** |  |  |  |  |  |  |  |
| No | Ref. | ND | ND |  | Ref. | ND | ND |
| Yes | 0.83 | (0.70-0.99) | ***0.0394*** |  | 0.82 | (0.69-0.98) | ***0.0273*** |
| **CCI score (Mean±SD), per score** | 1.08 | (1.00-1.17) | ***0.0492*** |  | 0.99 | (0.91-1.08) | 0.8377 |
| **Surgery** |  |  |  |  |  |  |  |
| No | Ref. | ND | ND |  | Ref. | ND | ND |
| Yes | 1.08 | (0.74-1.58) | 0.6819 |  | 1.02 | (0.70-1.49) | 0.9255 |
| CHM, Chinese herbal medicine; HR, hazard ratio; 95% CI, 95% confidence interval; ND, not determined; CCI, Charlson comorbidity index. | | | | | | | |
| Nasopharyngeal carcinoma (NPC) (ICD-9-CM-code: 147). | | | | | | | |
| CCI score (Mean±SD) was expressed as a continuous variable. | | | | | | | |
| Models adjusted for age, gender, CHM use, CCI score, and surgery. | | | | | | | |
| *P*-value (*p* < 0.05) was shown in bold italic font. | | | | | | | |

| **Table S12.** Cox proportional hazard models for NPC-related mortality in patients with advanced nasopharyngeal carcinoma (NPC) (stages 3+4) in Taiwan (cumulative days of CHM treatment ≧14 days within the first year of advanced NPC) | | | | | | | |
| --- | --- | --- | --- | --- | --- | --- | --- |
|  | **Crude** | | |  | **Adjusted** | | |
|  | **HR** | **(95% CI)** | ***P*-value** |  | **aHR** | **(95% CI)** | ***P*-value** |
| **Age (years old)** |  |  |  |  |  |  |  |
| Age<50 | Ref. | ND | ND |  | Ref. | ND | ND |
| 50≥Age<60 | 1.18 | (0.94-1.47) | 0.1591 |  | 1.17 | (0.93-1.47) | 0.178 |
| Age≥60 | 2.10 | (1.66-2.66) | ***<0.001*** |  | 2.12 | (1.64-2.74) | ***<0.001*** |
| **Gender** |  |  |  |  |  |  |  |
| Male | Ref. | ND | ND |  | Ref. | ND | ND |
| Female | 0.74 | (0.55-1.01) | 0.0545 |  | 0.75 | (0.55-1.02) | 0.0642 |
| **CHM use** |  |  |  |  |  |  |  |
| No | Ref. | ND | ND |  | Ref. | ND | ND |
| Yes | 0.84 | (0.70-1.01) | 0.0642 |  | 0.83 | (0.69-1.00) | ***0.0455*** |
| **CCI score (Mean±SD), per score** | 1.09 | (1.00-1.18) | ***0.0482*** |  | 1.00 | (0.91-1.09) | 0.9408 |
| **Surgery** |  |  |  |  |  |  |  |
| No | Ref. | ND | ND |  | Ref. | ND | ND |
| Yes | 1.09 | (0.74-1.60) | 0.655 |  | 1.03 | (0.70-1.51) | 0.8879 |
| CHM, Chinese herbal medicine; HR, hazard ratio; 95% CI, 95% confidence interval; ND, not determined; CCI, Charlson comorbidity index. | | | | | | | |
| Nasopharyngeal carcinoma (NPC) (ICD-9-CM-code: 147). | | | | | | | |
| CCI score (Mean±SD) was expressed as a continuous variable. | | | | | | | |
| Models adjusted for age, gender, CHM use, CCI score, and surgery. | | | | | | | |
| *P*-value (*p* < 0.05) was shown in bold italic font. | | | | | | | |
